# Functional conductive nanomaterials *via* polymerisation in nano-channels: PEDOT in a MOF†
†Electronic supplementary information (ESI) available. See DOI: 10.1039/c6mh00230g


**DOI:** 10.1039/c6mh00230g

**Published:** 2016-08-22

**Authors:** Tiesheng Wang, Meisam Farajollahi, Sebastian Henke, Tongtong Zhu, Sneha R. Bajpe, Shijing Sun, Jonathan S. Barnard, June Sang Lee, John D. W. Madden, Anthony K. Cheetham, Stoyan K. Smoukov

**Affiliations:** a Department of Materials Science and Metallurgy , University of Cambridge , Cambridge CB3 0FS , UK . Email: sks46@cam.ac.uk; b EPSRC Centre for Doctoral Training in Sensor Technologies and Applications , University of Cambridge , Cambridge CB2 3RA , UK; c Advanced Materials and Process Engineering Laboratory , University of British Columbia , Vancouver BC V6T 1Z4 , Canada; d Lehrstuhl für Anorganische Chemie II , Ruhr-Universität Bochum , Bochum 44801 , Germany

## Abstract

Reactions inside the pores of metal–organic frameworks (MOFs) offer potential for controlling polymer structures with regularity to sub-nanometre scales. We report a wet-chemistry route to poly-3,4-ethylenedioxythiophene (PEDOT)–MOF composites. After a two-step removal of the MOF template we obtain unique and stable macroscale structures of this conductive polymer with some nanoscale regularity.

## Abstract 2

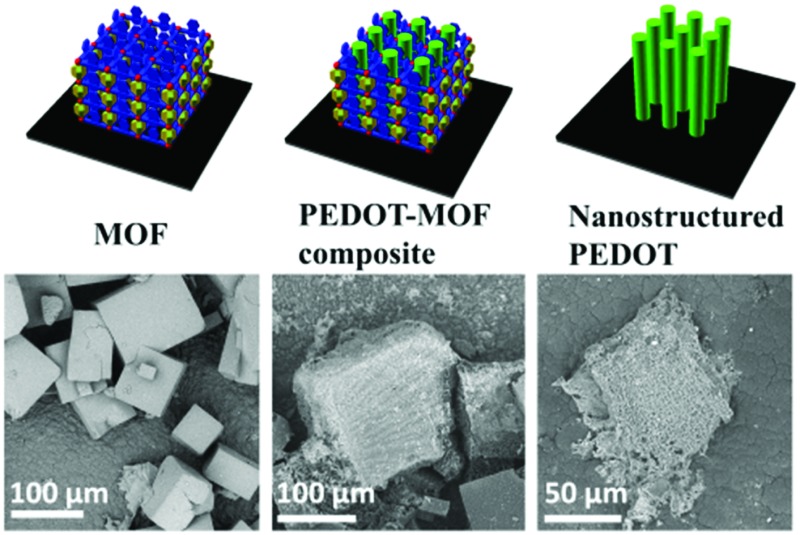
Poly(3,4-ethylenedioxythiophene) (PEDOT) is formed inside a metal–organic framework (MOF). MOF removal leads to sub-millimetre structures of the nanostructured conducting polymer.

Conceptual insights

Polymerisation in the confinement of nanometre pores is a recent endeavour with a number of challenges – both chemical (infusion of monomers in the pores and carrying out reactions), and mechanical (generating stresses, maintaining structural integrity). We demonstrate for the first time the synthesis of PEDOT in MOF's nano-channels, the resulting PEDOT–MOF composite, and removing the MOF template to yield macroscopic structures of nano-templated PEDOT. PEDOT is one of the most popular conducting polymers, due to its biocompatibility, thermal, chemical and electrochemical stability. The nanostructure we are able to achieve in PEDOT, will be useful for numerous applications such as sensors, actuators and supercapacitors. Moreover, rather than focusing on only the polymer itself, the method brings the idea of templating open-channel structures down to the single nanometre and molecular dimensions using MOFs. The methodology developed has yielded unique structures of nano-templated conducting polymer and is a novel manufacturing approach to multiple nanostructured materials.

Covalent organic frameworks (COFs)^1–3^ have opened up the potential for nanostructured polymers with further control on material properties due to high structural regularity. Alternative options for synthesising highly controlled polymer structures with long-range order include polymerisation in metal–organic frameworks (MOFs) or porous coordination polymers (PCPs),^4,5^ though there are synthetic difficulties in monomer infiltration and polymerisation within the MOF pores. Nonetheless, the MOF-templating approach has the potential to create nanostructured forms of a broad range of bulk materials from polymers^5^ to metals.^6^ Recent advances include electronically non-conductive nanostructured polymers from vinyl monomers^5,7,8^ (such as styrene, methyl methacrylate, vinyl acetate, divinylbenzene and acrylonitrile) reacting in MOFs and subsequent removal of the MOF template as well as formation of MOF–polymer structure by directly polymerising the organic ligands of the MOF *via* [2+2] cycloaddition reaction.^9,10^ Similar structures from conducting polymers would benefit the development of supercapacitors,^11–13^ sensor application,^14,15^ non-noble metal catalysis^16,17^ and fast actuation^18,19^ as a result of the high internal surface area and enhanced mass transport afforded. A few conducting polymer reactions have been reported in MOFs so far, including polypyrrole,^20^ poly(*N*-vinylcarbazole),^21^ polyaniline,^22^ polymethylpropylsilane^23^ and polythiophene,^24^ but self-standing structures of nanostructured conducting polymers are still required to be explored.

Though PEDOT^25–28^ has high electrical conductivity, chemical, electrochemical and thermal stability, high biocompatibility and good adhesion after deposition, nobody so far has described a PEDOT–MOF interpenetrating composite or the corresponding nanostructured polymer. PEDOT is the most widely available, stable and also one of the best characterised conducting polymers due to its use in poly(3,4-ethylenedioxythiophene)–poly(styrenesulfonate) (known as PEDOT:PSS) and other water-processable forms,^29–31^ so it has advantages relative to other polymerisations that have been attempted in MOFs.

Here we describe the formation of poly(3,4-ethylenedioxythiophene) (PEDOT) inside a large pore MOF template and subsequent removal to obtain sub-millimetre-sized nanostructured PEDOT (Fig. 1). We use as a template a MOF, Zn_2_(1,4-ndc)_2_(dabco) (1,4-ndc = 1,4-naphthalenedicarboxylate, dabco = 1,4-diazabicyclo[2.2.2]octane),^32^ hereafter referred to as MOFndc, infiltrated with the monomer EDOT and iron(iii) chloride (FeCl_3_, aq) as oxidant. We form PEDOT–MOF composites by oxidative polymerisation and then remove the MOF template to obtain conductive nanoporous PEDOT structures with similar size and shape to the composite crystals. We verify the compositions of all the structures using Raman spectroscopy, scanning electron microscopy (SEM) with energy-dispersive X-ray spectroscopy (EDS) and cathodoluminescence spectroscopy (CL), transmission electron microscopy (TEM) with electron energy loss spectroscopy (EELS), and powder X-ray diffraction (PXRD). The mechanical and electrical properties of the nanostructured PEDOT are also presented in this work. Nanostructured PEDOT on a conductive substrate has potential uses as an electrode for electrochemical sensors and supercapacitors.

**Fig. 1:**
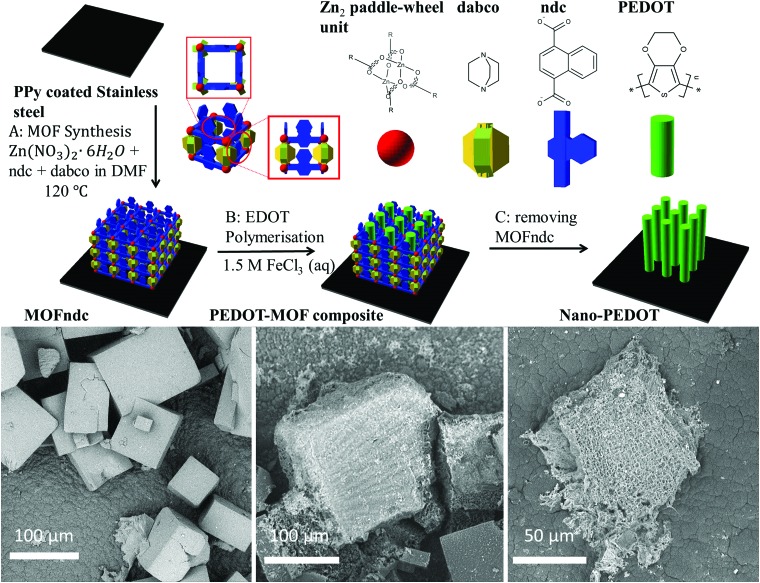
Schematic diagrams illustrate the experimental steps in sequence: synthesis of MOFndc on PPy coated stainless steel, chemical polymerisation of EDOT in MOFndc, and formation of nano-PEDOT after removing PEDOT. SEM images obtained with back-scattered electrons (BSE) reveal the morphologies of the products at the steps A–C. 3D schematics are created with open sourced software available from http://www.openscad.org/.

We grow MOF crystals on a conducting polymer substrate, so we can achieve electrical contact for the subsequently grown nano-PEDOT and minimise the chance for nanostructure delaminating from the substrate. EDOT is known to form excellent adhesion when it is polymerised onto a substrate.^27^ Additionally, the attachment of the PEDOT–MOF composite and nano-PEDOT structures on a relatively large substrate is also convenient for sample handling (*i.e.* no filtration or centrifugation required). Similar to a previous use of polyaniline for anchoring support,^22^ we used a polypyrrole (PPy) coated stainless steel for growing the MOF (ESI,† Fig. S1.4) with good adhesion to the substrate. This also leads to substrate adhesion of the polymer (nano-PEDOT) structures grown in the MOF templates.

The as-synthesised MOFndc (Fig. 1, left) consists of well-defined block shaped crystals with smooth surfaces and with dimensions of *ca.* 100 μm. The PXRD patterns (ESI,† Fig. S3.2) confirm their structural identity, matching data found in the literatures.^33,34^ MOFndc has the dicarboxylate ligands linked to Zn paddle-wheel units to form two-dimensional square grids. The layers are connected by dabco ligands at the lattice points.^35^ Meanwhile, Henke *et al.* ^34^ reported the anisotropic properties of this type of MOF, implying the anisotropic geometry of this structure type. Such MOF is also known to exhibit some degree of flexibility with regards to the pore dimensions upon taking up guest molecules.^34,36–38^ As shown in the PXRD patterns of MOFndc on a substrate (ESI,† Fig. S3.1–S3.5), the addition and removal of the guest molecules (here DMF or EDOT) from the MOF framework shift the peak positions, indicating a change in the structure of the framework due to the presence of the guests. Compared with MOFndc, the PEDOT–MOF composite (Fig. 1, middle) has a much rougher surface. The roughness is likely due to a combination of PEDOT that partially formed on the MOFndc surface and some degree of MOF dissolution and mechanical damage during polymerisation. The block shaped crystals of MOFndc are retained after polymerisation of PEDOT inside them. After removing the MOFndc, the nanostructured PEDOT retains approximately the same shape as the original MOF crystals (Fig. 1, right). The constant shape retention upon successive material replacements indicates that our synthesis for conducting polymers works (from the MOFndc to pure nano-PEDOT), similar to the results reported by Uemura *et al.* ^39^ for a MOF–polystyrene system *via* free radical polymerisation.

We confirm the successful growth of PEDOT inside MOFndc by characterising the structure, chemistry, and physical properties of the PEDOT–MOF composite. The 30 kV CL peak (ESI,† Fig. S4.2) shows a significant red shift from *ca.* 410 nm (pure MOFndc) to *ca.* 470 nm (PEDOT–MOF composite) indicating a reduction in the material's highest occupied molecular orbital – lowest unoccupied molecular orbital (HOMO–LUMO) gap, as the photons emitted *via* electron–hole recombination have lower energy (longer wavelength). The peak shift is not caused by FeCl_3_, as the CL peak for the FeCl_3_-incorporated MOF is at *ca.* 525 nm (ESI,† Fig. S4.4). The CL results imply a significant structural change or local chemistry change in MOFndc after the polymerisation. Some of the MOFndc crystals were not fully infiltrated with EDOT, resulting in a broad peak characteristic for a mixture of the pure MOF and the composite (ESI,† Fig. S4.3c). The PXRD pattern for the PEDOT–MOF composite also confirms the presence of MOFndc, as the major peaks for pure MOFndc are retained (ESI,† Fig. S3.1). Elemental analysis *via* SEM-EDS shows the MOFndc map is dominated by its Zn signal, with a S signal at a level similar to the background noise (Fig. 2a), whereas the MOF–PEDOT composite has identifiable Zn and S signals inferring the presence of MOFndc and PEDOT (Fig. 2b). After removing MOFndc using diluted hydrochloric acid (HCl, aq) and sodium hydroxide solution (NaOH, aq), we found sub-millimetre pieces of nanostructured polymer, confirming that the S signal in the composite was from PEDOT, not from EDOT. From Raman spectra (Fig. 2d), we observed peaks corresponding to both MOFndc and PEDOT for the composite. The peaks at *ca.* 1380 cm^–1^ (O=C–O vibration) and *ca.* 1590 cm^–1^ (C_α_ =C_β_ stretching) are due to ndc in the MOF;^40,41^ the peak at *ca.* 1420 cm^–1^ (C_α_ =C_β_ or C_α_ =C_β_(–O) stretching) is ascribed to PEDOT.^42,43^ Meanwhile, the peak emerging at *ca.* 700 cm^–1^ corresponds to the C–S feature of the thiophene.^42,43^ By combining the results above, the polymerisation of EDOT in MOFndc is confirmed. Unlike MOFndc and the PEDOT–MOF composite, nano-PEDOT gives a very weak CL signal – the polymer suffers from electron-induced structure damage^44^ when high-energy electrons (30 keV) are bombarding the sample to trigger cathodoluminescence. The similar effect was also observed for the as-synthesised PEDOT film (ESI,† Fig. S4.2). Though MOFs are highly susceptible to electron beam damage under TEM (200 kV),^45,46^ we noticed that the MOFndc is quite stable under CL characterisation (30 kV), *i.e.* the intensity and peak position are almost unaltered within a scanning period of a few minutes.

**Fig. 2:**
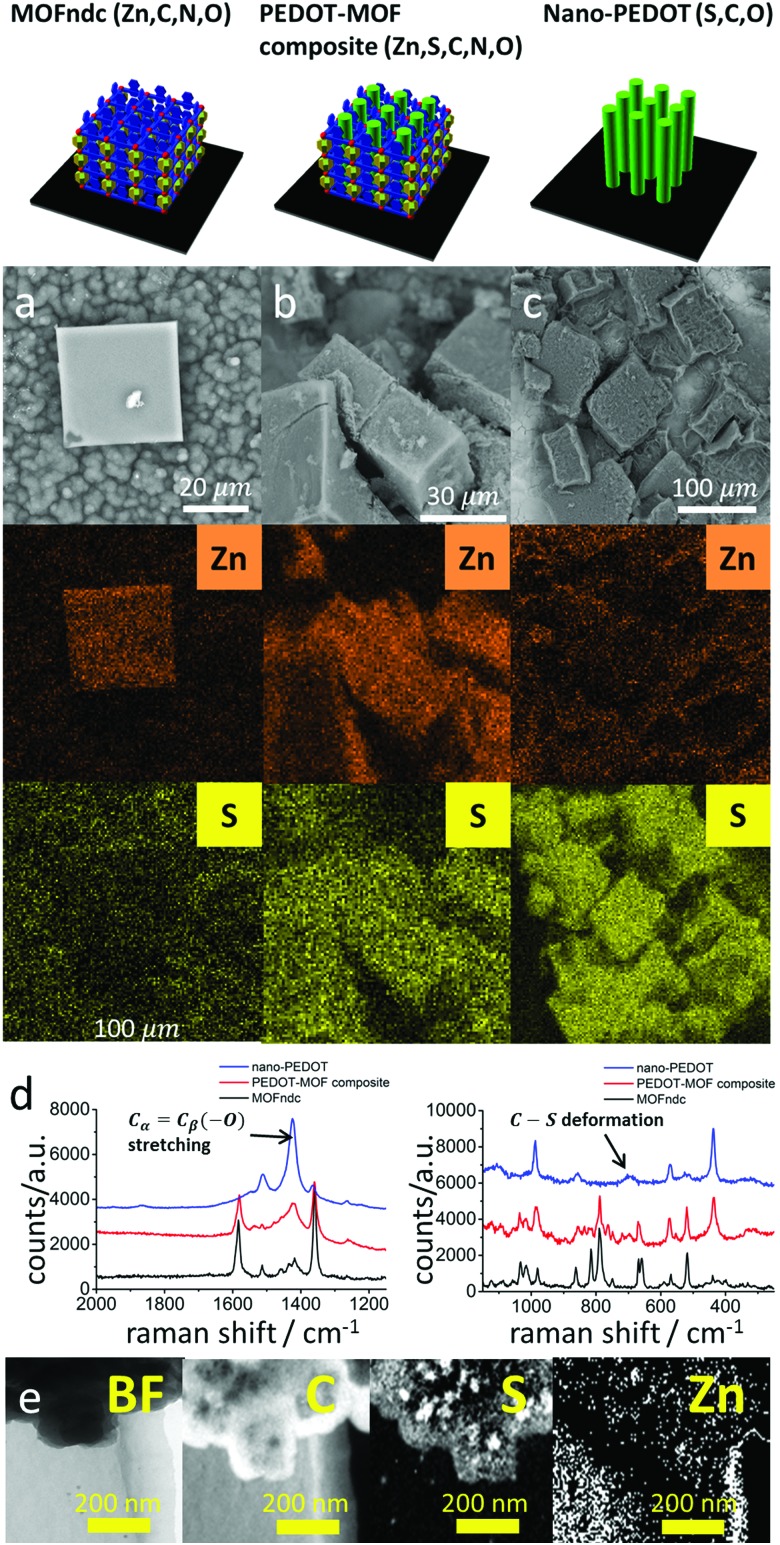
BSE-SEM images with EDS mappings of Zn and S for (a) MOFndc, (b) PEDOT–MOF composite and (c) nano-PEDOT. Raman spectra (d) extended scan (left) and static scan (right) around 700 cm^–1^ Raman shift. (e) Energy filtered transmission electron microscopy (EFTEM) images with EELS mappings of C (K-edge, 284 eV), S (L-edge, 165 eV) and Zn (L-edge, 1020 eV).

Nano-PEDOT structures were obtained by removing the MOFs in acid and base. SEM-EDS elemental analysis (Fig. 2c) shows a drop-off in Zn intensity, and S is the dominant signal. EFTEM-EELS mapping (Fig. 2e), which is more applicable to lighter elements, confirms the presence of C and S in the nano-PEDOT, and shows no appreciable Zn signal above the background noise. Raman spectra also confirm the presence of nano-PEDOT (Fig. 2d and ESI,† Fig. S4.12). The overall spectrum shape from extended scan (ESI,† Fig. S4.12) matches well with the spectrum reported in the literatures, with a single strong peak at *ca.* 1420 cm^–1^ for symmetric C_α_ =C_β_(–O) stretching in PEDOT (also shown in Fig. 2d).^42,43^ The peak at *ca.* 700 cm^–1^ for the C–S ring deformation in PEDOT can also be clearly seen in Fig. 2d. Furthermore, the peak at *ca.* 790 cm^–1^ for the cage-breathing mode of dabco^47^ disappears, reflecting the removal of MOFndc.

On some facets (Fig. 3a and b) the nano-PEDOT structures show rough surface morphologies with no preferred direction. In contrast, other facets (Fig. 3c and d) reveal highly directional fibre-like morphology with a typical fibre diameter of *ca.* 40 nm. These aligned fibres are likely formed by collapse of much smaller polymer fibrils in the same direction. High resolution TEM image reveals some structural alignments (ESI,† Fig. S5.1), which is different from the amorphous polymer structure observed for bulk PEDOT (ESI,† Fig. S5.4). Such aligned structures are likely to be PEDOT fibrils that are formed *in situ* in the one-directional nano-channels separated by the non-reactive pore walls (ESI,† Fig. S5.2).^48^ The results are comparable to those reported by Distefano *et al.* ^4^ for MOF–templated polystyrene. Another possibility for the aligned structure found by TEM, however, is iron oxide precipitate from the FeCl_3_ used in the reaction. Although iron oxide could have much sharper signal,^49^ we have not conclusively eliminated its possibility. The 3D nano-PEDOT structures are likely to be due to strong lateral non-covalent interactions between the fibrils. Removal of the MOF results in a decrease of the actual inter-chain spacing of fibrils, which also leads to an increase of the conductivity of nano-PEDOT, compared to the PEDOT–MOF composite.

**Fig. 3:**
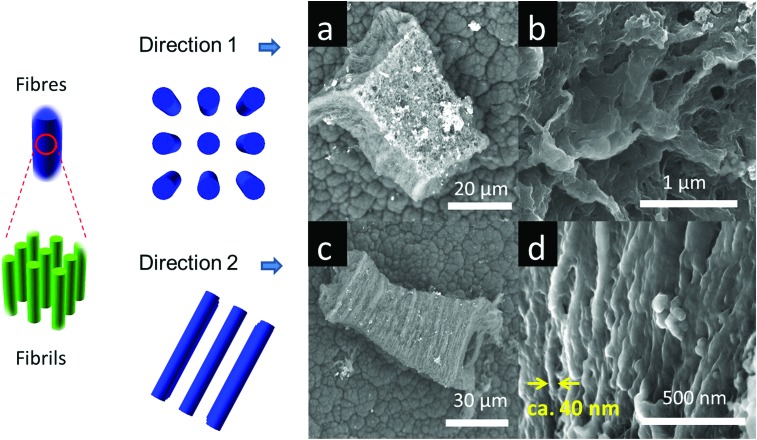
PEDOT fibres (in blue) consists a bundle of fibrils (in green) that are formed in the MOF paralleled nano-channels. Since the PEDOT fibers are mostly aligned, SEM shows only rough surface in some orientations as (a) and (b), whereas it reveals aligned fibre-like morphology in other orientations as (c) and (d).

Fig. 4a and b show significant difference in the mechanical properties of MOFndc and PEDOT. The Young's modulus of MOFndc was measured as *E* = 3.2 ± 0.9 GPa, averaging over the indentation depths between 200 nm to 900 nm (Fig. 4b). The lower modulus compared with MOFndc single crystals (7.4 ± 0.2 GPa^34^) is likely to be due to a loss of guest molecules (DMF in this case). Meanwhile, since the crystals were tested as deposited on the substrate, they are likely not perfectly normal to the indenter axis resulting the lower modulus. Further nanoindentation results performed for MOFndc are provided in ESI,† Fig. S6.1. The Young's modulus of nano-PEDOT itself was found to be 0.50 ± 0.17 GPa, which is an order of magnitude lower than the MOFndc framework, and also lower than the value reported for bulk PEDOT (*ca.* 2 GPa^50,51^), implying that it is more porous than bulk PEDOT. However, the current synthesis method produces very limited amount of nano-PEDOT, which constrains the application of further characterisation techniques such as PXRD and gas adsorption measurement. Next stage will address these questions to consolidate the materials' features.

**Fig. 4:**
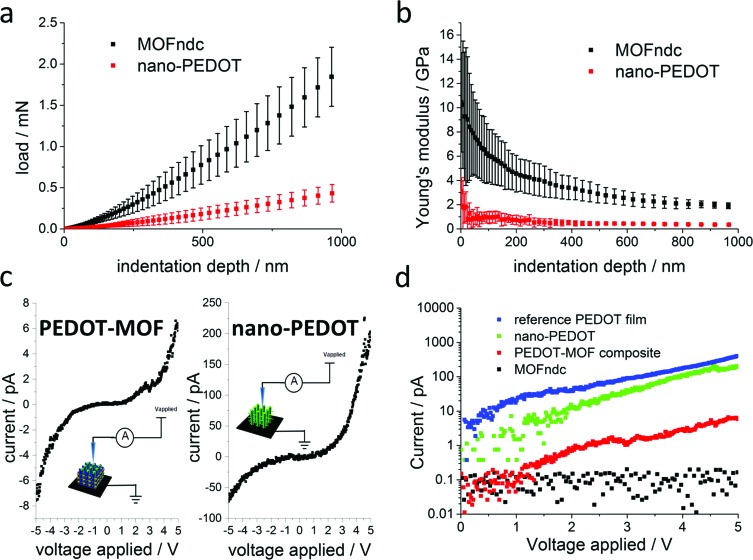
Nanoindentation experiments for MOFndc crystals with rectangular cross-sections and nano-PEDOT (over 4 indents on each sample, error bars representing the standard deviations): (a) load *versus* displacement and (b) Young's modulus *versus* indentation displacement depth. Conductive atomic force microscopy (AFM) experiments: (c) *I*–*V* curves for PEDOT–MOF composite and nano-PEDOT; (d) current measured in log 10 scale for MOFndc, PEDOT–MOF composite, nano-PEDOT and as-synthesised PEDOT film. All the samples were immobilised on the PPy-coated stainless steel.

Achieving a nanostructured conducting polymer is an unmet challenge so we measured the current–voltage (*I*–*V*) curves of the materials synthesized (Fig. 4c and d) to confirm the presence of conducting material. Our PEDOT–MOF composite and nano-PEDOT show semiconducting behaviours. The shapes of the curves are influenced by the mobility of the charge carriers, which is voltage dependent.^52^ Compared with the *I*–*V* curve for MOFndc (an insulator), the current for the PEDOT–MOF composite is at least one order of magnitude higher (Fig. 4d) when the applied voltage is above +2 V or below –2 V. Meanwhile, a further two orders of magnitude increase in current is noticed on the *I*–*V* curve of nano-PEDOT, which is marginally lower than the PEDOT film synthesized in the same condition without the MOF template. The high conductivity of the PEDOT structures implies high molecular weight achieved during the PEDOT polymerisation.^53^ The procedures used for washing the unpolymerised EDOT were developed for much larger structures, so by the scaling of diffusion, these small structures are likely to be washed out of left-over monomers. Previously, apart from some special MOFs that exhibit intrinsic conductivity,^54,55^ electrical conduction was achieved by incorporating small molecules such as 7,7,8,8-tetracyanoquinododimethane (TCNQ) into the MOF^56–58^ or by forming a conducting polymer within the MOF.^59^ In our case, we achieve a stable conductive composite by polymerising PEDOT within the MOF comparable with the PEDOT–MOF system reported by Le Ouay *et al.*,^60^ but we are also able to remove the MOF to obtain a free-standing nano-PEDOT polymer structure. Some of the high conductivity is likely due to leftover Fe counterions (3% in weight percent, approximate quantification from EDS measurements, ESI,† Fig. S4.8). Further incorporation with different salts or counterions within MOFs, such as direct synthesis of PEDOT:PSS in a MOF with sufficiently large pores, could be used to increase or tune the conductivity of these structures. Radical polymerisation is regarded as an efficient mass-scale production method, but challenges in carrying it out in nano-channels lead to poor control on nanostructures and properties.^5^ Channel-promoted polymerisation with a redox reaction^5^ is an alternative approach that is even less understood but with the potential for better control of polymer nanostructures and properties due to easier control of reaction kinetics. Further improvements on our process of polymerisation of PEDOT in a MOF framework could open up possibilities for studying the fundamentals of such reactions in confined spaces. The key questions will be around: (i) the migration (*e.g.* diffusion) of small species (*e.g.* ions, radicals and small molecules), (ii) the polymerisation mechanisms (the initiation process and the propagation process), (iii) the interactions between the guests (*e.g.* monomers and polymers) and the nano-channels, and (iv) the mechanism for removing the MOF template and its consequences (*e.g.* potential collapse).

Such an approach has unrivalled potential for synthesising a broad range of functional polymers with structural regularity approaching molecular level. Compared with conventional templates, such as confinement inside aluminium oxide membranes and colloidal particles crystals,^61,62^ MOFs or similar frameworks have even smaller regular features. Interpenetrating polymers in MOFs or similar materials could result in highly controlled aligned structures even in the sub-nanometre regime. This could promote the development of numerous applications in fast actuation, electrochemical sensing, redox reactions for supercapacitors, and non-noble metal catalysis due to their high surface area and the potential mitigation of diffusion limitations into structures.

## Experimental methods

### Materials

HCl (aq, 37% w/w), NaOH, lithium perchlorate (LiClO_4_), dabco, 1,4-naphthalenedicarboxylic acid (H_2_ndc), zinc nitrate hexahydrate (Zn(NO_3_)_2_·6H_2_O), *N*,*N*-dimethylformamide (DMF), FeCl_3_ and EDOT were purchased from Sigma-Aldrich and used as received. Pyrrole was also ordered from Sigma-Aldrich and distilled at *ca.* 160 °C before use. Acetonitrile was purchased from Fisher Scientific. AISI 403 stainless steel was cut into 10 × 10 mm^2^ square pieces for the substrates.

### Synthesis

MOFndc was synthesised on the PPy coated stainless steel substrate (explained in ESI†) by immersing the substrate into the pre-filtered MOF precursor solution, which consists of 0.42 mmol Zn(NO_3_)_2_·6H_2_O, 0.42 mmol ndc, 0.21 mmol dabco and 10 ml DMF. The solution together with the substrate was sealed in a Teflon-lined autoclave and kept at 120 °C for 48 hours in an oven. The autoclave was then removed from the oven and cooled under ambient conditions. The as-grown MOFndc was stored in fresh DMF at room temperature. It was dried at 150 °C and soaked in pure EDOT for 4 hours. The sample was treated at 100 °C to remove the EDOT outside, and then immersed in the excess FeCl_3_ (aq) oxidant solution (pH ∼ 2) for 15 h. The PEDOT–MOF composite was rinsed with methanol several times to remove the non-polymerised monomer and also excess oxidant solution (FeCl_3_) until no colour is observed in the washing solvent.^63^ To isolate nano-PEDOT from the MOFndc, it was immersed into pH ∼ 2 HCl (aq) solution for 1 day followed by pH ∼ 4 HCl (aq) solution for another day to prevent precipitation from residual Fe ions as well as to gradually remove Zn_2_ paddle-wheel unit and dabco. It was then transferred to water for 2 hours followed by pH ∼ 12 NaOH (aq) solution for more than 1 day to remove the rest of MOFndc. The sample was eventually rinsed with water and dried under ambient conditions.

### Characterisation

BSE-SEM images and EDS mapping were acquired on a Phenom ProX Desktop microscope with an accelerating voltage of 5–15 kV for BSE-SEM images and 15 kV for EDS mapping. High-resolution SEM images were obtained using a FEI Nova NanoSEM™ with a secondary electrons detector with 10 kV acceleration voltage. HR-TEM images and EELS were performed using 200 kV FEI Tecnai™ F20 with a field emission gun. Raman spectra were obtained using a silicon-calibrated Renishaw Ramascope-1000 with a 633 nm red laser source. Powder X-ray diffraction patterns were collected on a Bruker D8 ADVANCE with 2*θ* from 4° to 55° and a step size of 0.05°. CL studies were performed at room temperature and 30 kV in a Philips XL30 SEM equipped with a Gatan MonoCL4™ system. *I*–*V* curves were measured in the contact mode using Pt/Ir-coated silicon probes on a Veeco Dimension 3100 atomic force microscope with a linear current amplifier module with a range from 1 pA to 1 μA. Nanoindentation was performed at ambient conditions using an MTS NanoIndenter® XP. A sharp three-sided pyramidal Berkovich indenter (tip radius ∼100 nm) was aligned normal the MOF. With a dynamic continuous stiffness measurement (CSM) mode, as reported in previous work,^64,65^ Young's moduli and hardnesses were deduced using the Oliver and Pharr method.^66^

## Conclusions

In summary, we show the synthesis of a nanostructured conducting polymer (nano-PEDOT) in the nano-confinement of a MOF template, and subsequent removal of the template by sequential acid–base treatment. We characterise the identity of both the MOF–polymer composite and then the nano-structured polymer materials by a wide variety of analytical techniques, including EDS, Raman spectroscopy and others. The MOF–polymer composite shows at least an order of magnitude higher conductivity than the MOF, whereas the nano-PEDOT conductivity approaches that of bulk PEDOT. Porosity of the nano-PEDOT results in a Young's modulus that is only a fraction of that of bulk PEDOT. These flexible and conductive materials have potential applications in sensing, actuation, and energy conversion and storage.

## Author contributions

The manuscript was written through contributions of all authors. All authors have given approval to the final version of the manuscript. SKS, AKC, SH, SRB and JSL initiated the project. TW, MF, SKS, JDWM and AKC conceived the idea about polymerizing EDOT in MOF. TW, MF, TZ, SRB, SS, JSB and JSL performed the experiments, characterizations and measurements. SH fitted and analysed the PXRD patterns. TW and MF wrote the manuscript instructed by SKS, AKC and JDWM with input from all the authors.
