# Supplementary material for: Functional conductive nanomaterials via polymerisation in nano-channels: PEDOT in a MOF
Source: Mater Horiz. 2016 Aug 22;4(1):64–71. doi: 10.1039/c6mh00230g (PMC5361137; doi:10.1039/c6mh00230g)
Supplement: Supplementary file 1 [file MH-004-C6MH00230G-s001.pdf]

Electronic Supplementary Material (ESI) for Materials Horizons.
This journal is © The Royal Society of Chemistry 2016

Supporting Information

Conductive Functional Nanomaterials via Polymerisation in Nano-channels: PEDOT in a MOF

*Tiesheng Wang, Meisam Farajollahi, Sebastian Henke, Tongtong Zhu, Sneha R. Bajpe, Shijing Sun, Jonathan S. Barnard, June Sang Lee, John D.W. Madden, Anthony K. Cheetham, and Stoyan K. Smoukov**

Contents

1. Additional Experimental 1
  1.1 Polypyrrole deposition on Stainless steel 1
  1.2 MOF Stabilized on PPy Coated Stainless Steel 2
2. Thermal Analysis of EDOT-loaded MOF 4
3. XRD analysis 6
4. Optical Images and Additional Characterizations on Materials Chemistry 9
5. Additional TEM and SEM characterizations 16
6. Supporting Results for Nanoindentation 19

1. Additional Experimental

1.1 Polypyrrole deposition on Stainless steel

Polypyrrole (PPy) was polymerised on a stainless steel by electrochemical deposition. In this method, deposition solution is a composition of 0.05 Lithium perchlorate ($LiClO_4$), 0.06 M distilled pyrrole and 1% (v/v) distilled water in acetonitrile. A piece of stainless steel mesh served as a counter electrode and a AISI 403 stainless steel substrate was used as a working electrode. Deposition was done at -20 ^oC by applying a constant current (0.125 mA/cm^2) for 8 hours. During the polymerisation process, polymerised pyrrole or PPy is deposited on the stainless steel substrate as working electrode. The one side of the stainless steel was masked with Kapton© tape. After deposition, the PPy film was rinsed with pure acetonitrile for 3 times followed by rinsing with DI water. The sample was dried under ambient condition.

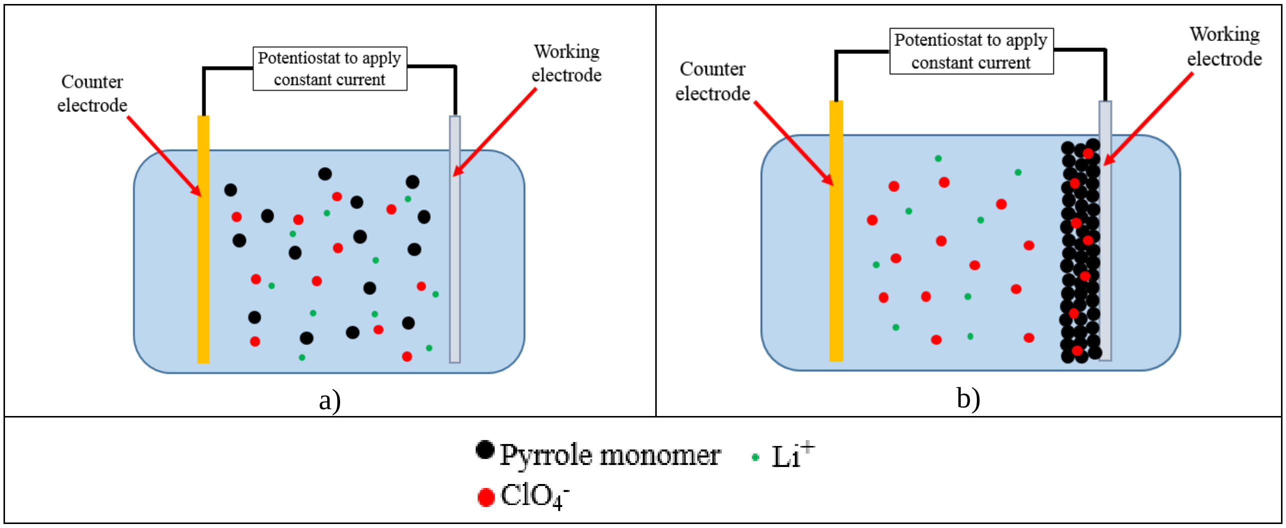

Suppl. Fig. S1.1: Setup for deposition of the polypyrrole a) before applying current b) after applying current.

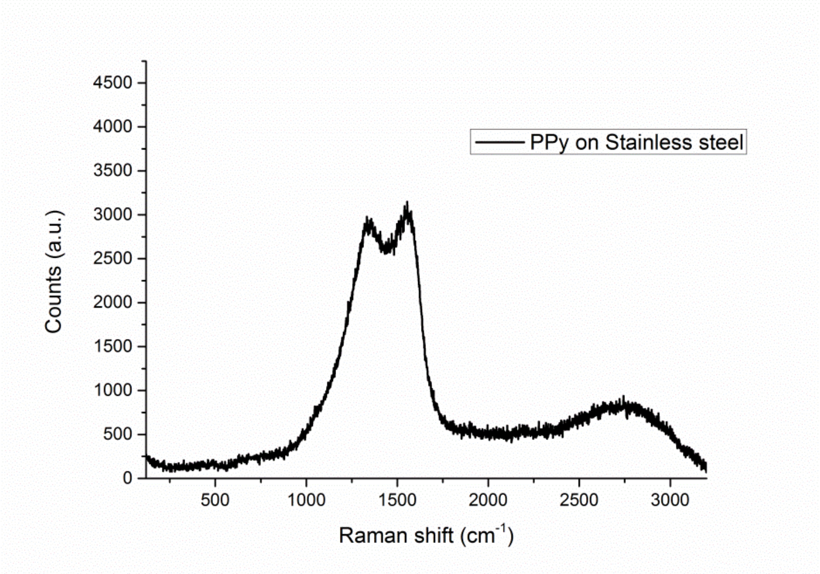

Suppl. Fig. S1.2: Raman extended scan on PPy coating .

1.2 MOF Stabilized on PPy Coated Stainless Steel

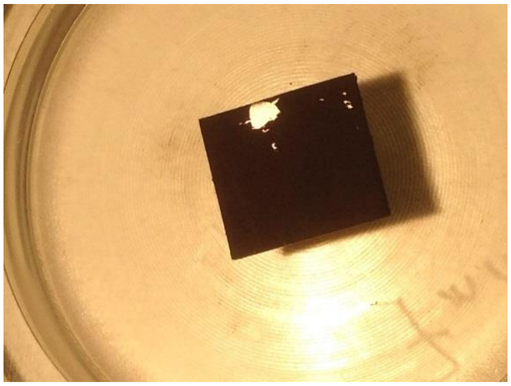

Suppl. Fig. S1.3: PPy coating synthesised on a stainless steel substrate via the described electrochemical method.

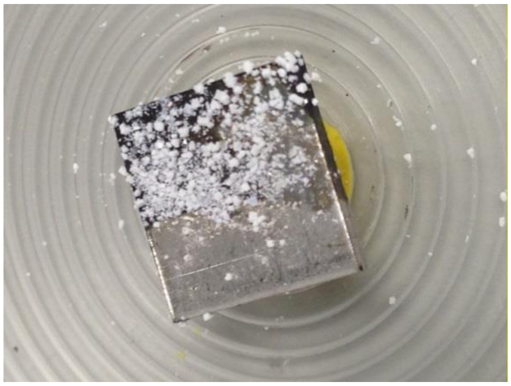

Suppl.Fig. S1.4: Most MOF crystallites will be localized at the PPy coated part of the stainless steel substrate after MOF synthesis in an autoclave.

2. Thermal Analysis of EDOT-loaded MOF

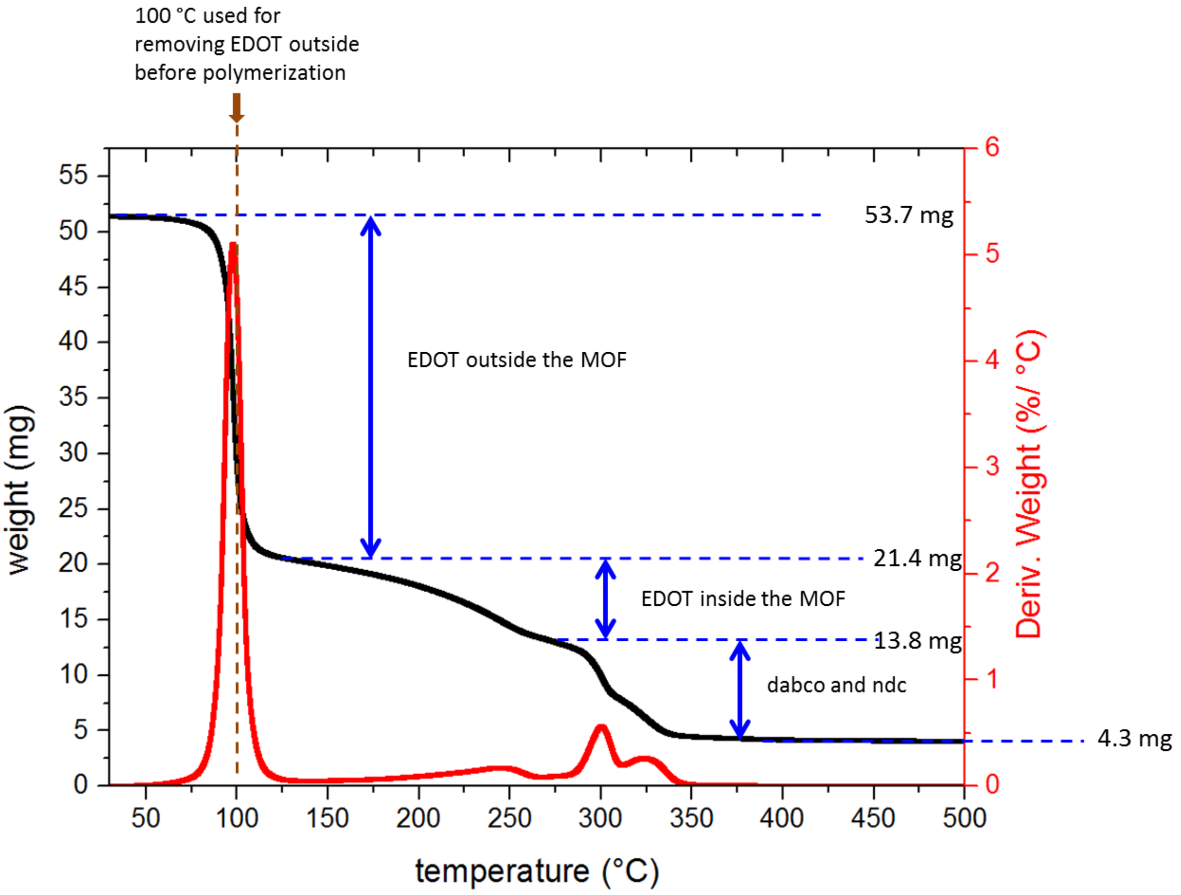

Suppl. Fig. S2.1: TGA of EDOT-loaded MOFndc.

Dynamic heating rate thermogravimetric analysis (TGA) was performed with a TA Instruments Q500 TGA. The dynamic heating rate TGA is an improved TGA method which can give high-resolution mass-loss profile with improved separation for mass-loss steps.[1]

Though the MOFndc used here has copper as metal linker instead of zinc, the MOFs have very similar unit cell parameters with almost equal monomer absorbed.[2] The monomer absorbed determined by copper-based MOFndc is comparable with that for zinc-based MOFndc. The TGA for pure MOFndc can be found in [3], which shows that residual DMF can be removed at ca. 150 ^{o}C.

Suppl. Fig. S2.1 shows the TGA for a MOFndc sample collected after being immersed in EDOT monomer. The sample was thermally treated at 150 ^{o}C to remove residual DMF before soaking the EDOT. TGA steps indicate different chemical reactions and/or physical transitions with unique activation energies. Starting from room temperature, the first step was due to vaporisation of extra EDOT outside the MOF. Such vaporisation occurs mostly at ca. 100 ^{o}C indicated by the peak for deriv. weight (weight loss for incremental temperature increase). Therefore, for the real synthesis, EDOT-infiltrated MOF was treated at 100 ^{o}C to remove the monomer outside.

The second step in TGA was due to desorption of EDOT inside the MOF. These two processes have different activation energies, as EDOT is likely to require higher activation to overcome interactions within the nano-channels than just evaporation. The second step is between ca. 125 ^{o}C and ca. 270 ^{o}C is used to estimate the amount of EDOT absorbed by the MOF. Above ca. 270 ^{o}C, the MOF is considered to start decompose.

Since the molecular weight of the MOFndc ($Cu_2(1,4\text{-ndc})_2(dabco)$) unit cell is 667.55 g/mol, the mass of EDOT that is loaded into 1 mol MOF unit cell can be obtained from the weight loss in the second step, which is 8.1 mg shown in **Suppl. Fig.** S2.1:

$$667.55 \times \frac{7.6}{13.8} \approx 367.6 \text{ g}$$

As EDOT has a molecular weight of 142.18 g/mol, the number of EDOT loaded in a MOF unit cell is:

$$\frac{367.6}{142.18} \approx 2.6$$

The number is comparable with the number for styrene (2.2) and methyl methacrylate (2.3) monomers absorbed by the same copper-based MOFndc and those number for zinc-based MOFndc (2.1 styrene monomer and 2.3 methyl methacrylate monomer) [2] . Both monomers show the shape retention after polymerising the monomers and removing the MOF.[4]

3. XRD analysis

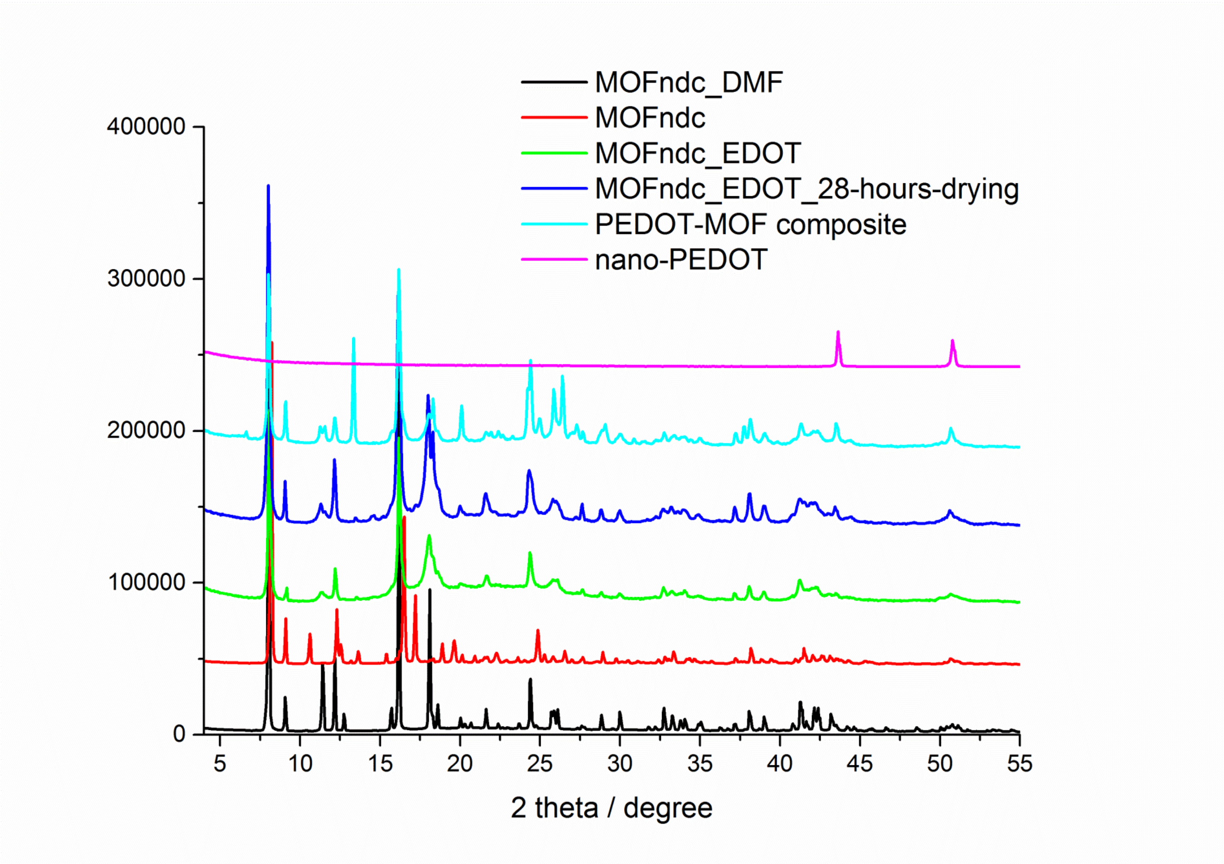

Suppl. Fig. S3.1: PXRD (powder X-ray diffraction) overview for MOFndc/PEDOT system.

No significant peak is found in the PXRD pattern for nano-PEDOT. One of the reasons is that the amount of sample with the nano-scaled alignment is insufficient. From the TEM images, we observed that the majority of nano-PEDOT structures show no chain alignment. Without perpendicular structures connecting parallel fibrils (acting as crosslinkers[5]) to keep them aligned, the structure is likely to suffer collapse upon removing the MOF template.

The as prepared (DMF containing) MOFndc crystallises in the tetragonal space group *P*4/*mbm*.[6] A Pawley fit (done with the TOPAS academic V5 program package) to the diffraction data of the MOFndc sample prepared on a PPy coated stainless steel substrate is in excellent agreement with the literature data (see **Suppl. Fig.** S3.2).

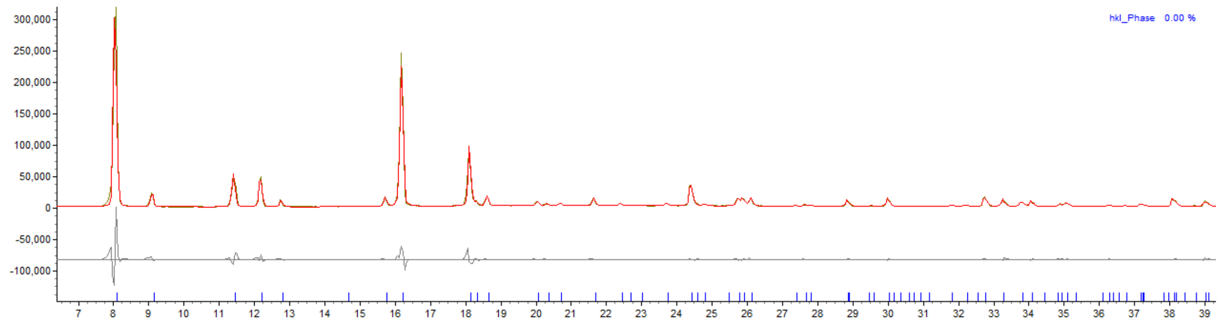

Suppl. Fig. S3.2: Pawley fit to the diffraction pattern of as prepared (DMF containing) MOFndc grown on a PPy coated stainless steel substrate (space group *P*4/*mbm*, $a = 15.4546(8)$ Å, $c = 9.6714(9)$ Å; $R_p = 9.28\%$, $R_{wp} = 13.42\%$, $R_{exp} = 1.16\%$. Experimental, calculated and difference patterns are shown in brown, red and grey, respectively. Positions of allowed Bragg peaks are shown as blue tick marks.

Upon drying in air (partial loss of DMF guest molecules) the material changes its structure as indicated by the unsuccessful Pawley refinement of the corresponding PXRD pattern using the initial unit cell parameters and space group (**Suppl. Fig.** S3.3). However, the pattern can be fit tolerable to a distorted orthorhombic unit cell in space group *Pmmm* (**Suppl. Fig.** S3.4). This indicates that the square channels of MOFndc distort to rhomb-shaped channels upon air-drying, as has been reported for various other MOFs of this family.[7,8]

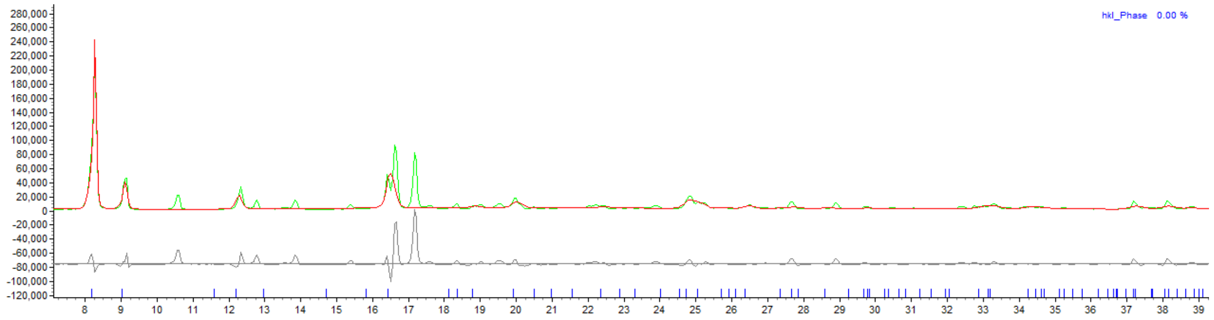

Suppl. Fig. S3.3: Attempt of a Pawley fit to the diffraction pattern of air-dried MOFndc (grown on a PPy-coated stainless steel substrate) in the space group *P*4/*mbm* and unit cell parameters similar to literature values. The data cannot be fitted satisfactory in this space group, indicating a significant distortion of the structure as a consequence of (partial) loss of DMF upon drying in air. Experimental, calculated and difference patterns are shown in green, red and grey, respectively. Positions of allowed Bragg peaks are shown as blue tick marks.

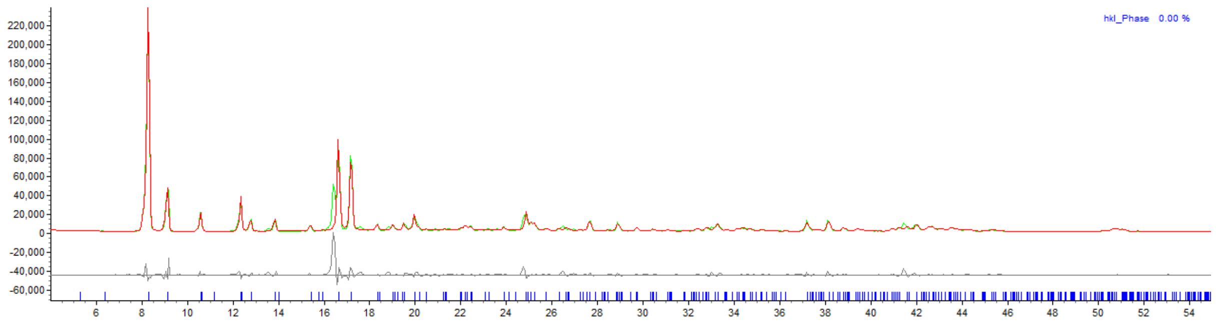

Suppl. Fig. S3.4: Attempt of a Pawley fit to the diffraction pattern of air-dried MOFndc (same pattern as in **Suppl. Fig.** S3.3) in the orthorhombic space group *Pmmm* (a = 16.653(4) Å, b = 13.828(5) Å, c = 9.660(3) Å; R_p = 10.75%, R_{wp} = 18.32%, R_{exp} = 1.05%). A reflection at approx. 16.4 degrees 2theta could not be fit with the chosen unit cell and space group, and may originate from an impurity. Experimental, calculated and difference patterns are shown in green, red and grey, respectively. Positions of allowed Bragg peaks are shown as blue tick marks.

Evidently, there are significant changes to the structure upon (partial) loss and/or exchange of the guest molecules. The MOF hosting EDOT exhibits much lower crystallinity than the DMF-containing as-prepared sample. The pattern features significantly broader peaks but could be fit satisfactory using a simple tetragonal unit cell in the high symmetry space group *P*4/*mmm* (**Suppl. Fig.** S3.5).

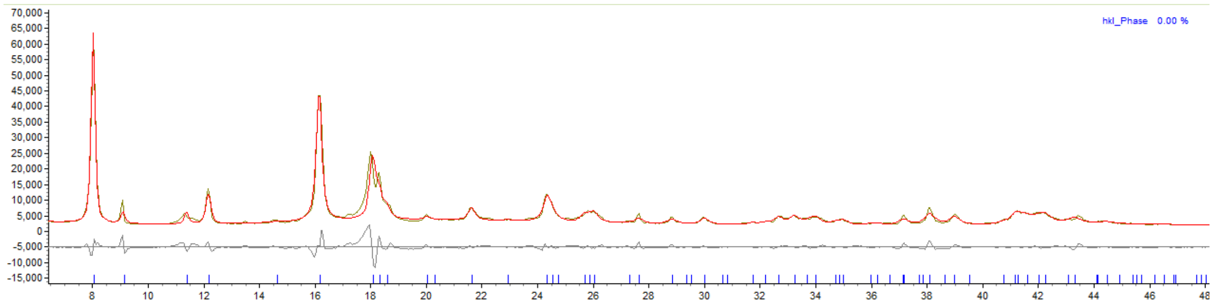

Suppl. Fig. S3.5: Attempt of a Pawley fit to the diffraction pattern of EDOT-containing MOFndc (grown on a PPy-coated stainless steel substrate; space group *P*4/*mmm*, a = 10.956(3) Å, c = 9.672(4) Å; R_p = 7.49%, R_{wp} = 11.15%, R_{exp} = 1.44%). Experimental, calculated and difference patterns are shown in dark yellow, red and grey, respectively. Positions of allowed Bragg peaks are shown as blue tick marks.

The diffraction pattern of the PEDOT-containing MOFndc (after polymerisation of EDOT in the pores) could not be indexed in one of the unit cells used earlier. There are several additional peaks in the pattern, which have not been present in the as-prepared MOFndc (**Suppl. Fig.** S3.1). Indexing the diffraction pattern with the pattern indexing routine in the TOPAS academic program package was unsuccessful as well. This leads to the conclusion that the pattern of the PEDOT-MOF composite does not correspond to a single phase. It rather may be that several structurally slightly different phases of the composite coexist. However, the major peaks for MOFndc are still approx. at the same positions as for the as-prepared material, which indicates that structurally intact MOFndc is still present after the polymerisation of EDOT. The additional peaks may correspond to decomposition products of MOFndc due to the treatment with acidic $FeCl_3$ (aq) solution.

4. Optical Images and Additional Characterizations on Materials Chemistry

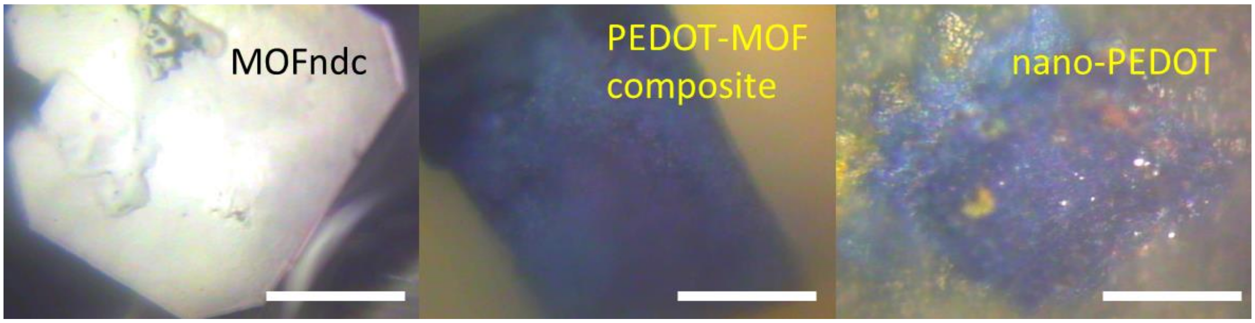

Suppl. Fig. S4.1: Optical images for MOFndc, PEDOT-MOF composite and nano-PEDOT. All the scale bars represent 50 µm.

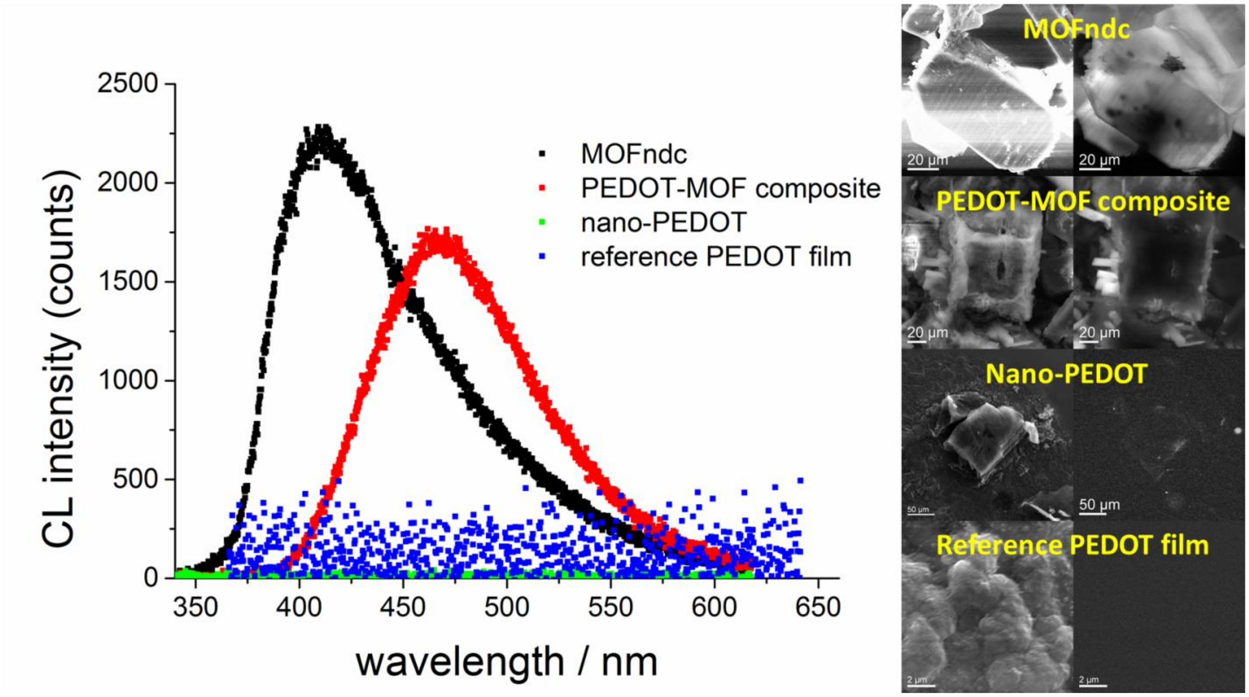

Suppl. Fig. S4.2: SEM images (left column), 30 kV cathodoluminescent (CL) images (right column) and the corresponding CL spectra for MOFndc, PEDOT-MOF composite, nano-PEDOT and a reference PEDOT film made with the same condition. All the samples are on the PPy-coated stainless steel substrate. Samples were rinsed with methanol for a few time to remove FeCl3. The peak's redshift from ca. 420 nm (black) to ca. 470 nm (red) further proved that PEDOT is inside the MOF leading to the local chemistry change.

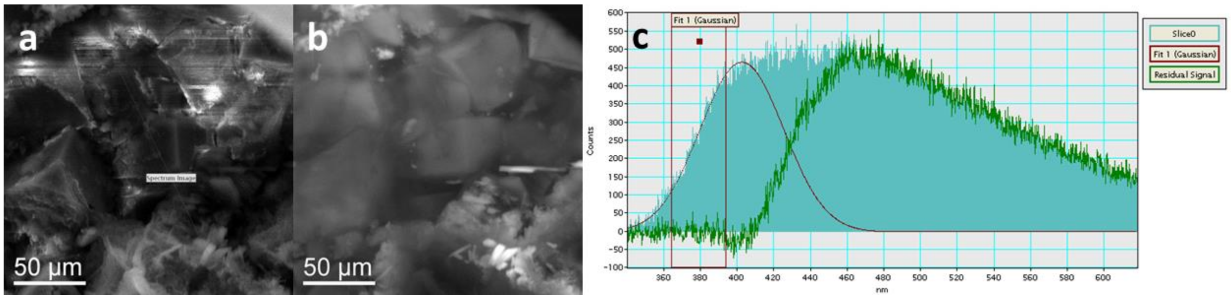

Suppl. Fig. S4.3: CL line scan from the region for MOFndc and PEDOT-MOF composite mixture and the region dominated by the composite: (a) SEM image, (b) CL image, (c) the CL spectrum for the region containing both MOFndc (400-420 nm) and the PEDOT-MOF composite (ca. 470 nm).

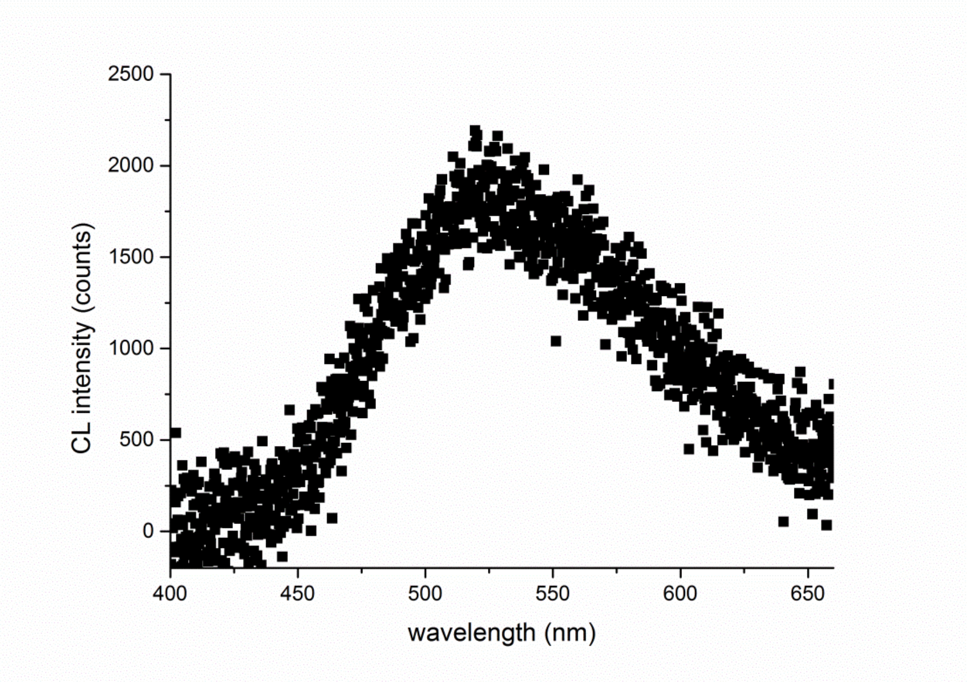

Suppl. Fig. S4.4: CL spectrum for the MOFndc incorporated with $FeCl_3$. $FeCl_3$ incorporating in the MOF leads to the peak at ca. 525 nm, which is different compared with the peak for PEDOT in MOF.

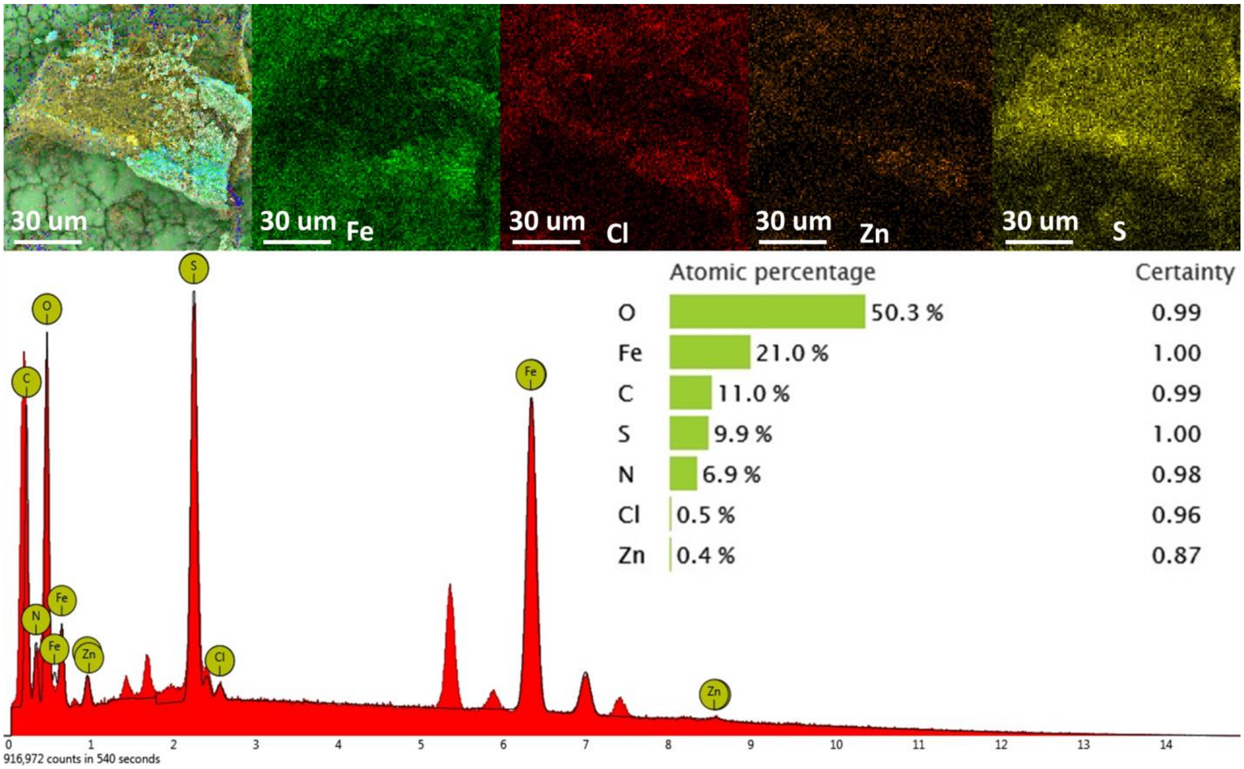

Suppl. Fig. S4.5: EDS (energy-dispersed X-ray spectroscopy) analysis on semi-washed sample. Left part of the sample reveals the nano-PEDOT features whereas right part of the sample is dominated by the residual from MOFndc and $FeCl_3$.

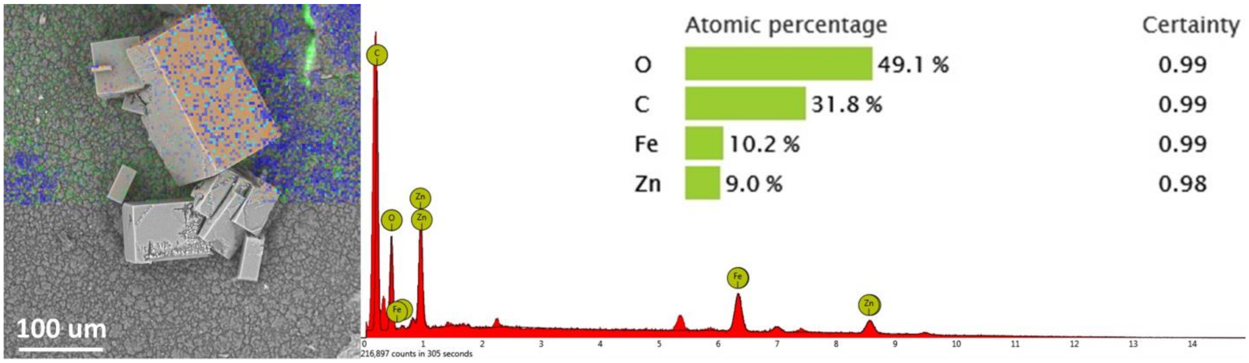

Suppl. Fig. S4.6: SEM(BSE)-EDS analysis MOFndc .

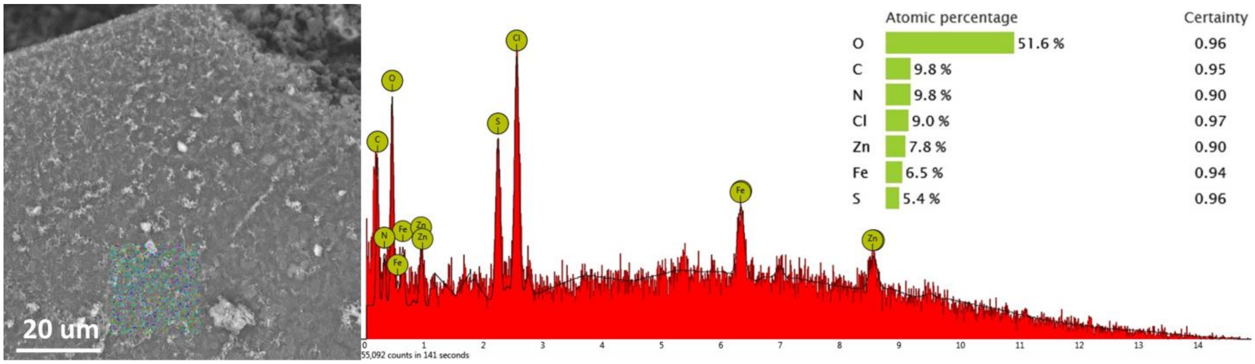

Suppl. Fig. S4.7: SEM(BSE)-EDS analysis on PEDOT-MOF composite .

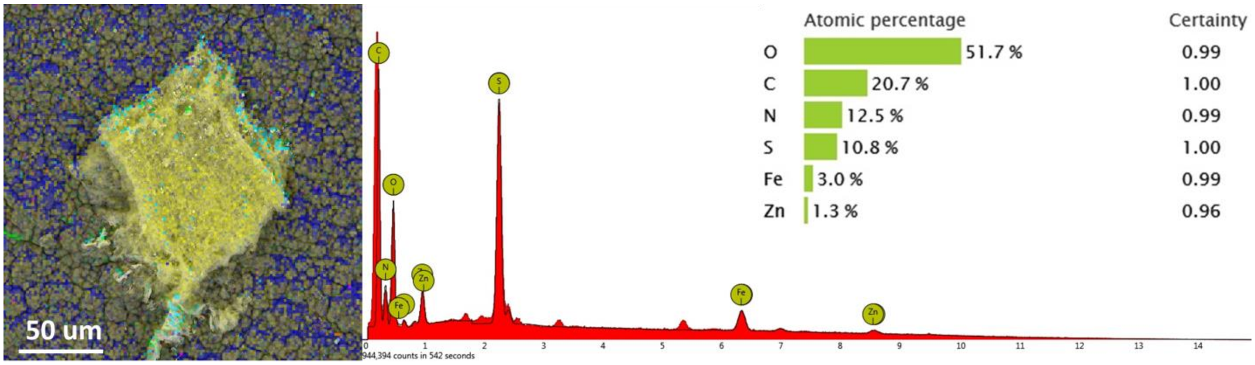

Suppl. Fig. S4.8: SEM(BSE)-EDS analysis on nano-PEDOT .

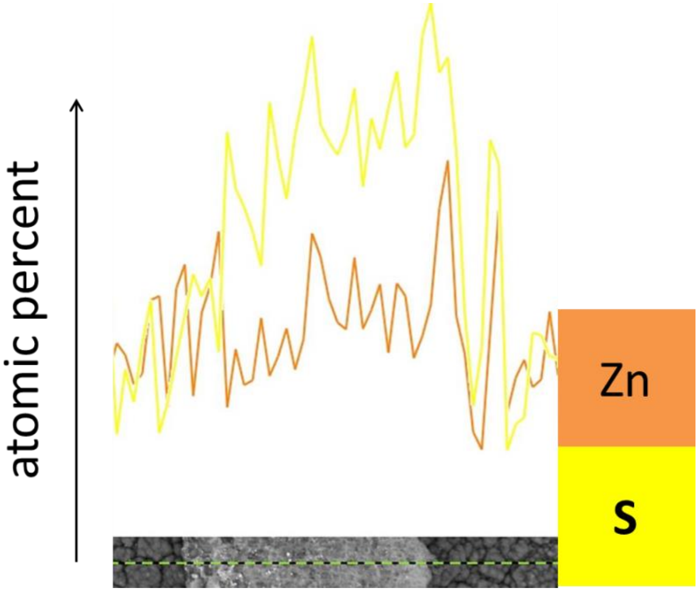

Suppl. Fig. S4.9: BSE-EDS line profile on nano-PEDOT .

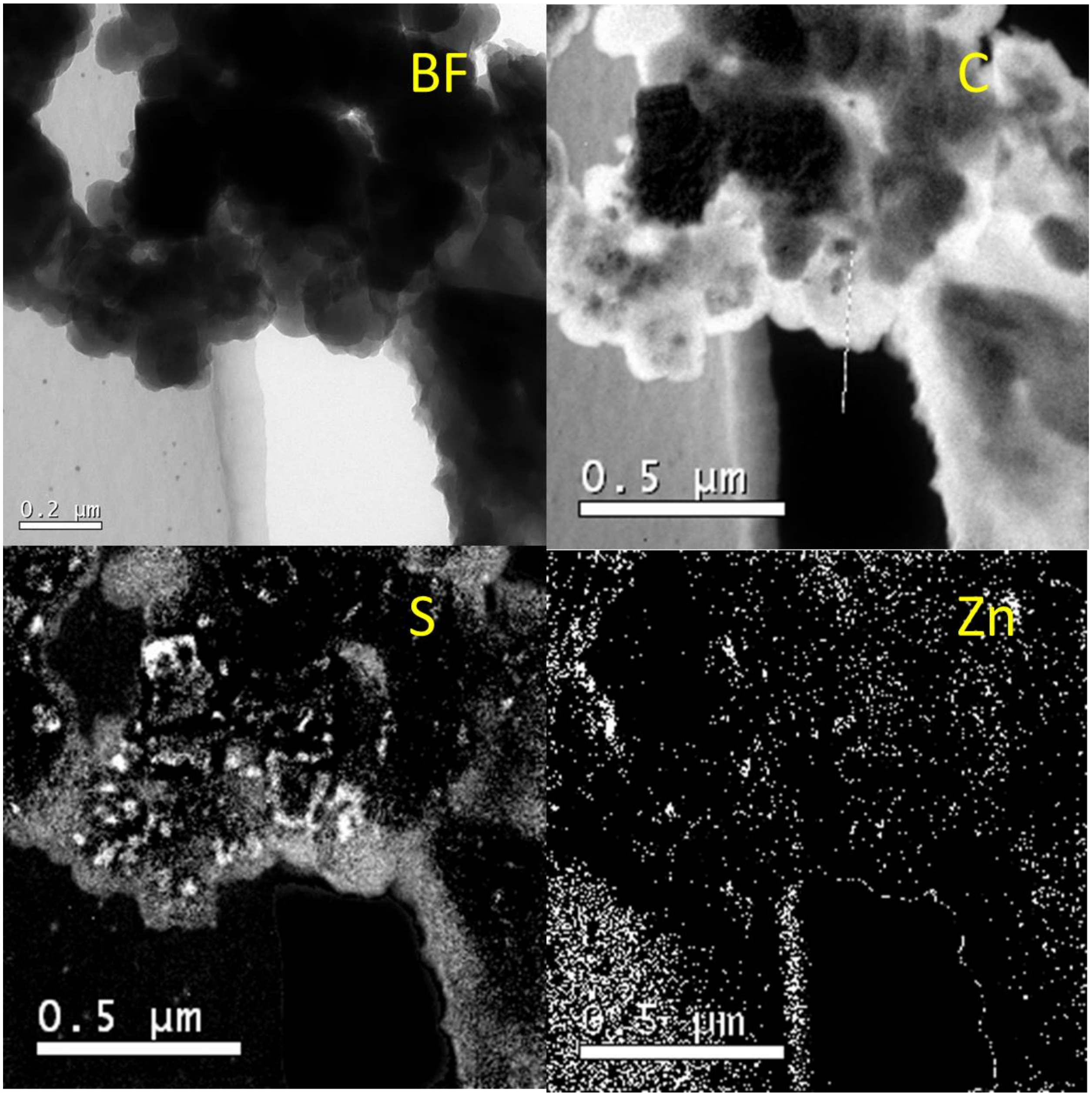

Suppl. Fig. S4.10: Full EFTEM-EELS (energy-filtered transmission electron microscopy – electron energy loss spectroscopy) mappings.

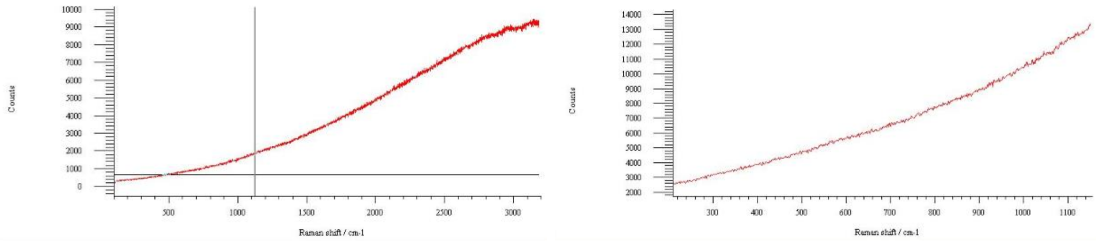

Suppl. Fig. S4.11: Raman spectra (extended LHS and static RHS) on glass support (background).

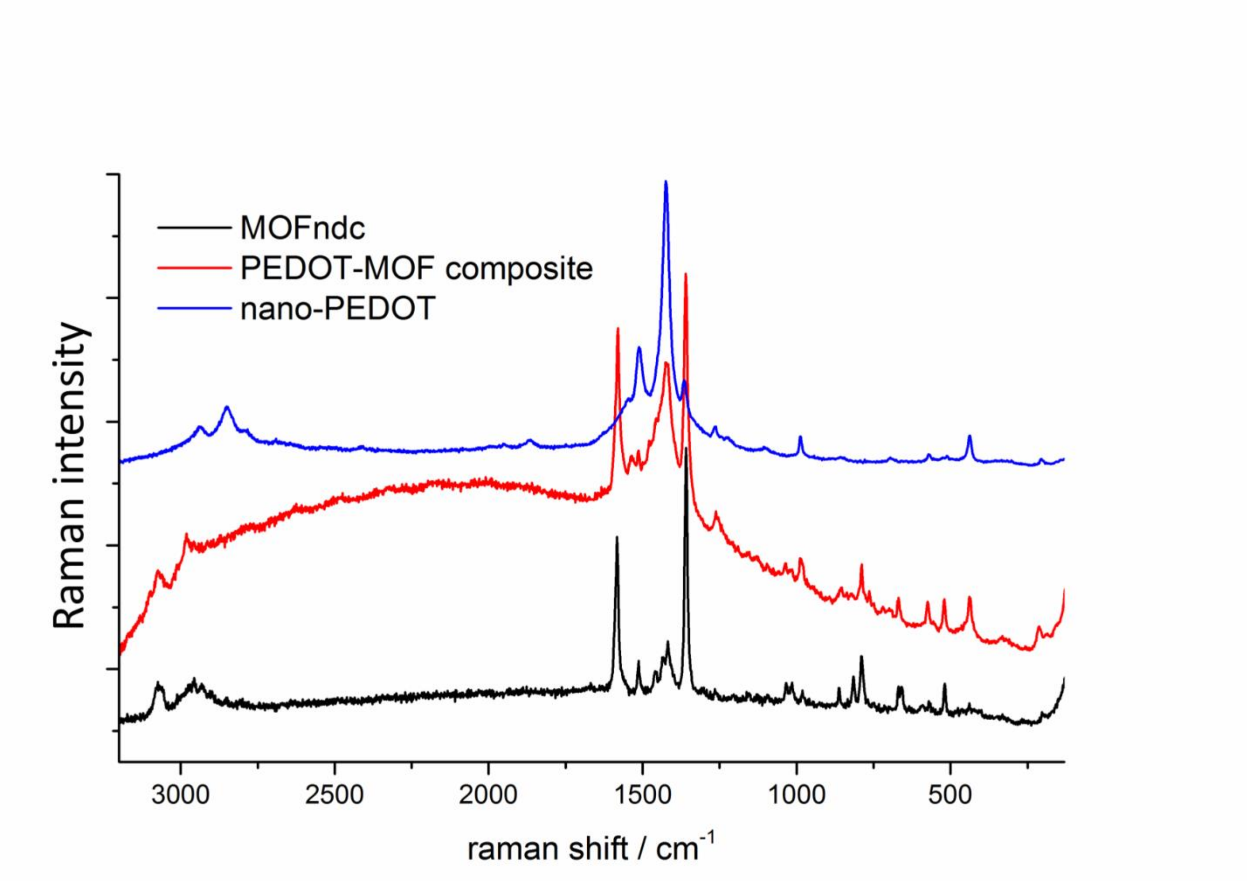

Suppl. Fig. S4.12: Extended Raman scanning for MOFndc, PEDOT-MOF composite and nano-PEDOT.

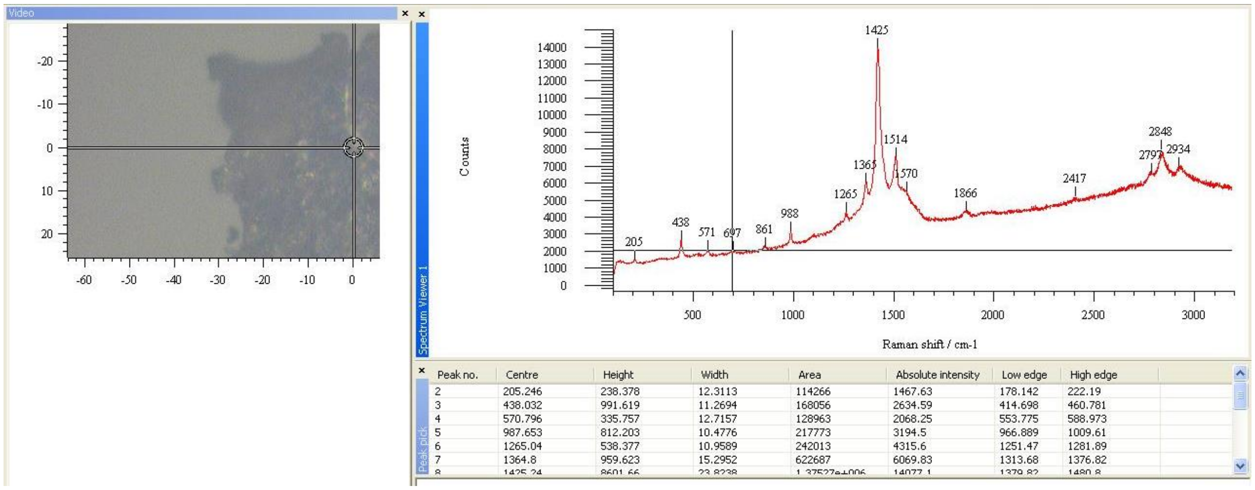

Suppl. Fig. S4.13: Raman spectrum for nano-PEDOT in the extended scanning mode. The optical image of the targeted sample is shown on the right hand side.

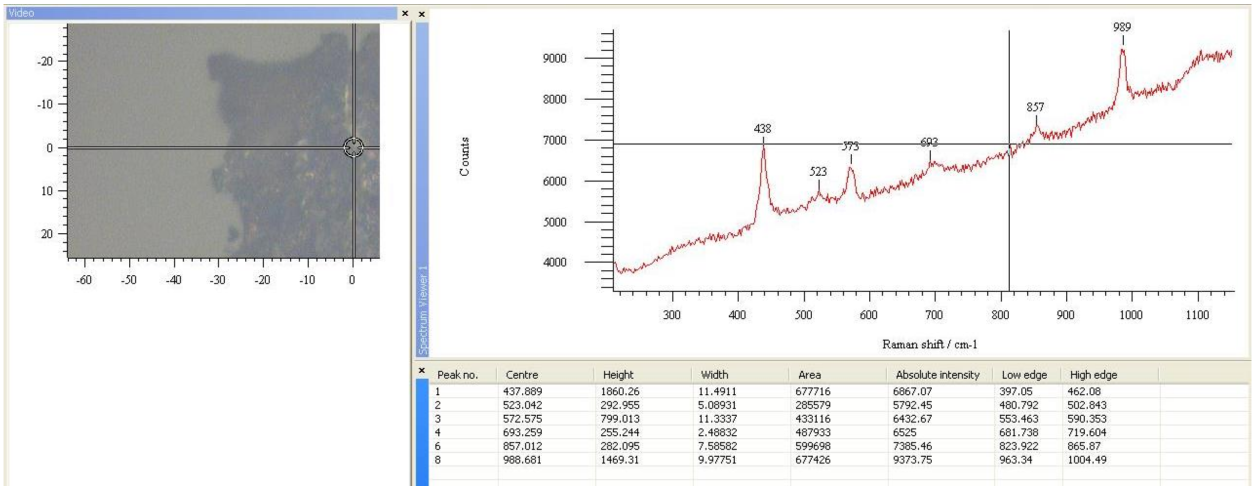

Suppl. Fig. S4.14: Raman spectrum for nano-PEDOT in the static scanning mode. The optical image of the targeted sample is shown on the right hand side.

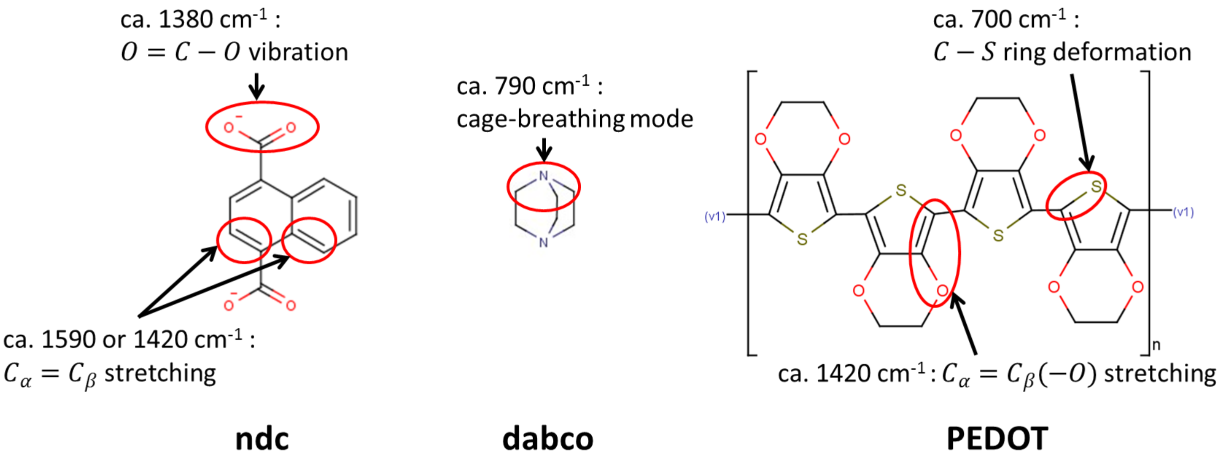

Suppl. Fig. S4.15: Raman peaks and their origins for ndc and dabco in MOFndc and PEDOT based on the literatures.[9–13]

5. Additional TEM and SEM characterizations

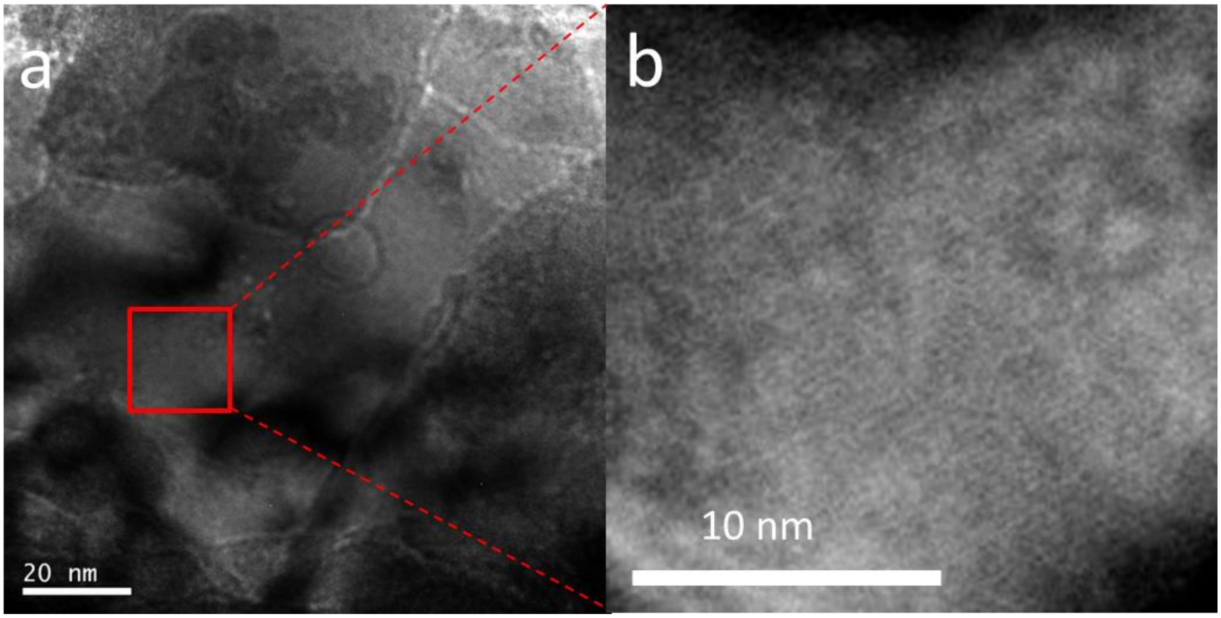

Suppl. Fig. S5.1: TEM images for nano-PEDOT indicating the position taken for the high resolution TEM figure in the main content.

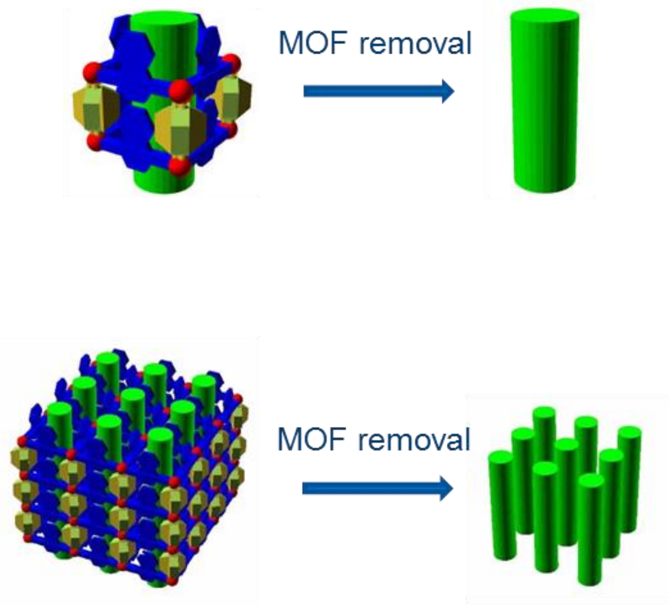

Suppl. Fig. S5.2: One possible origin of the alignment observed from the TEM: EDOT polymerisation in open-channels of MOFndc. The parallel nanochannels formed by 4 ndc ligands (window is 5.7 Å × 5.7 Å^2) can accommodate the *in situ* polymerisation of EDOT leading to the polymer chain alignment. The TEM shows a 5 Å inter-chain spacing (**Suppl. Fig.** S5.1), which is consistent with laterally-aggregated fibrils after removal of the MOF. Previously, such highly aligned nanostructures were observed by Distefano *et al.*[5] for MOF-templated polystyrene, but we demonstrate such alignment in a MOF-templated conducting polymer for the first time. However, the chemistry identity of the fibrils is still an open question.

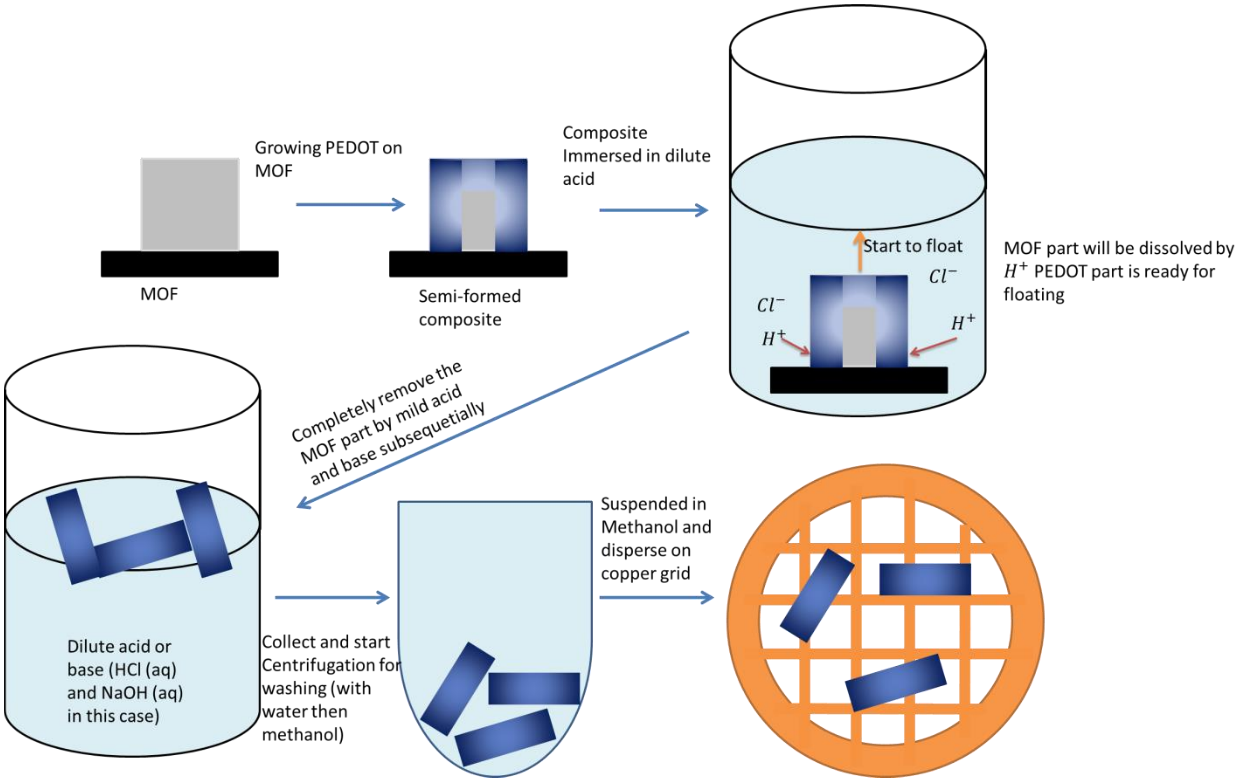

Suppl. Fig. S5.3: Steps for preparing the TEM sample

TEM sample preparation procedures for nano-PEDOT are illustrated in **Suppl. Fig.** S5.2. Briefly, the PEDOT-MOF composite was placed in HCl (aq) followed by NaOH (aq). Those composite with PEDOT only formed in some part (mostly close to the surface) of MOF was attacked by the acid and the base gradually. Since those semi-formed composites have limited PEDOT connection to the substrate, they started to detach the substrate after the MOF part was sufficiently dissolved. The detached material was collected by centrifugation and immersed in fresh acid and base respectively to completely remove the MOF. It was then rinsed with water and methanol. The prepared sample was suspended in methanol and loaded on the TEM copper grid by drop casting.

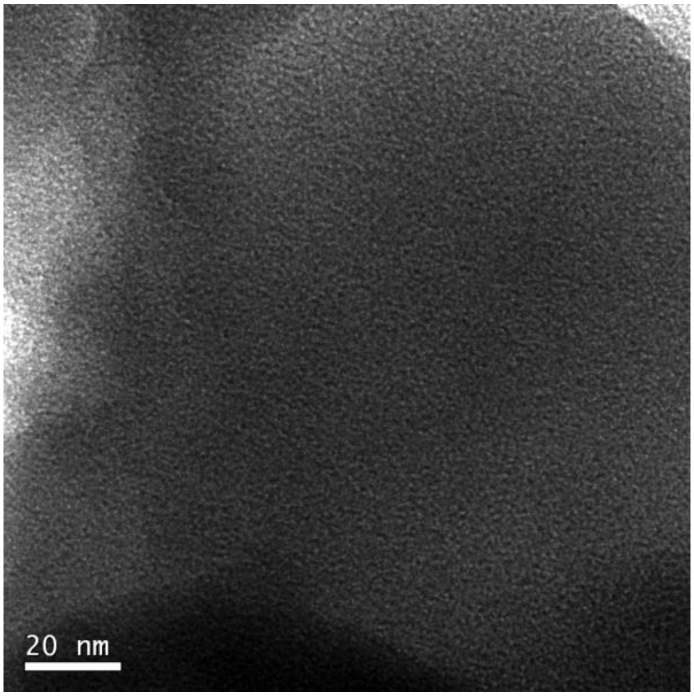

Suppl. Fig. S5.4: TEM image for normal bulk PEDOT (amorphous).

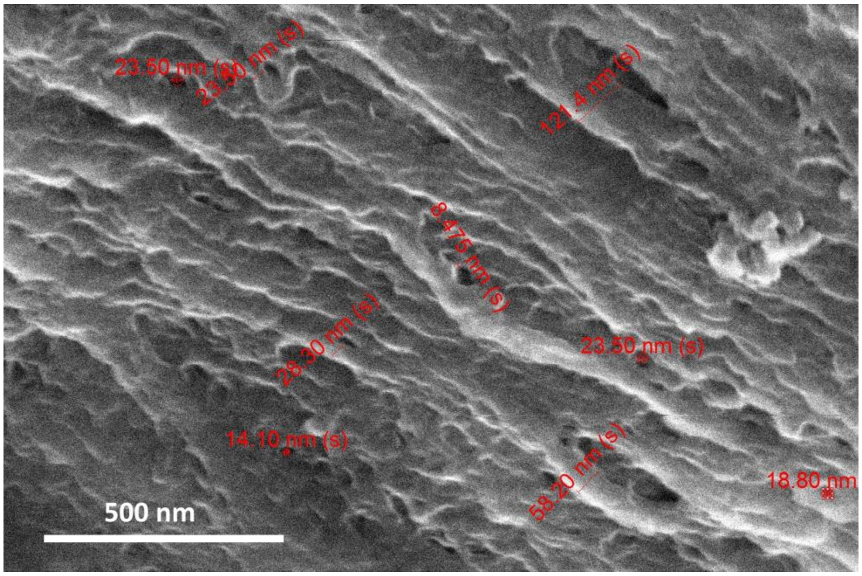

Suppl. Fig. S5.5: SEM image showing more submicron-scale features.

6. Supporting Results for Nanoindentation

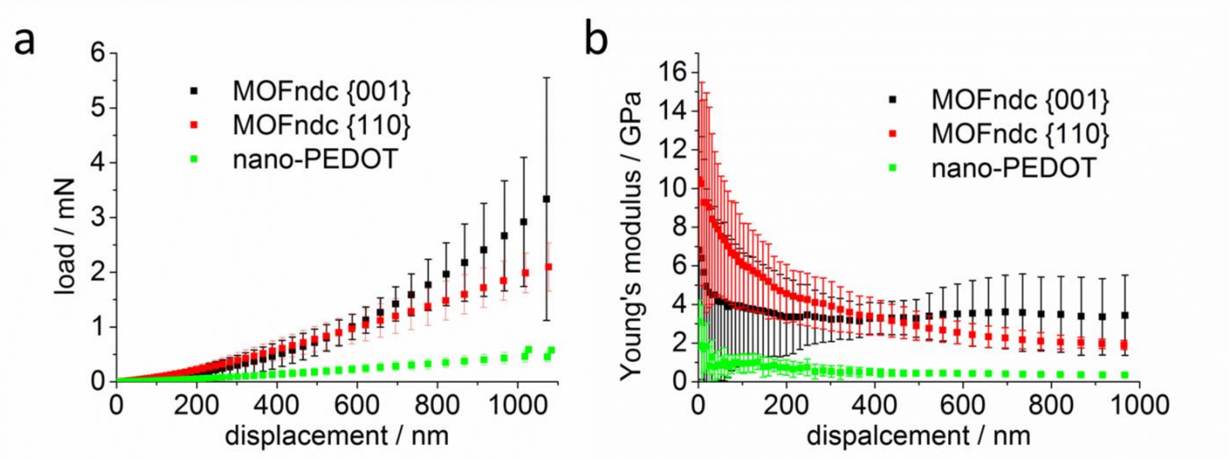

Suppl. Fig. S6.1: Nanoindentation experiments for MOFndc along {110} (rectangular cross-section) and {001} (square cross-section) and nano-PEDOT (over 4 indents on each sample, error bars representing the standard deviations): (a) load versus displacement and (b) Young's modulus versus indentation displacement depth. The Young's moduli of MOFndc were measured as E_{110} = 3.2±0.9 GPa and E_{001} = 3.3±0.8 GPa, averaging over the indentation depths between 200 nm to 900 nm.

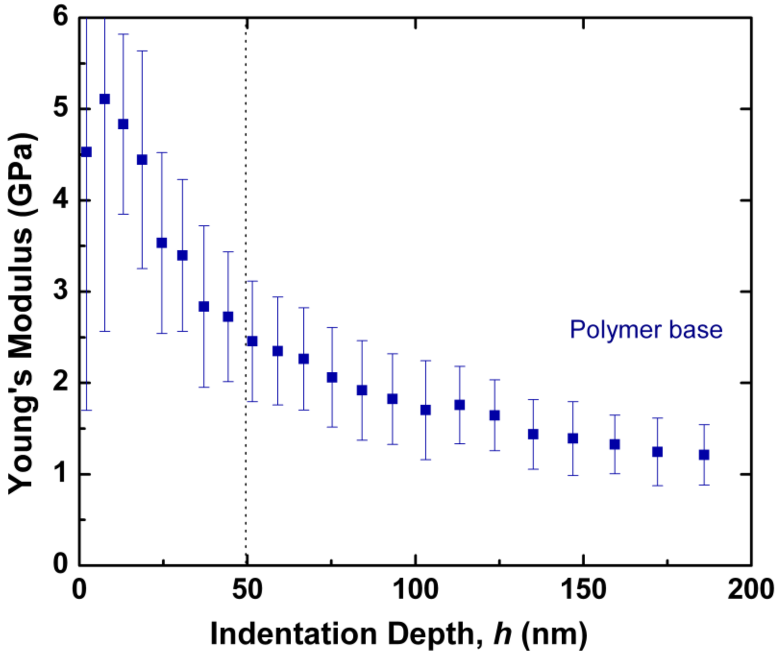

Suppl. Fig. S6.2: Nanoindentation results for PPy coating on stainless steel: Young's Modulus = 1.9±0.5 GPa, averaging over 5 indents from indentation depth of 50 to 150 nm.

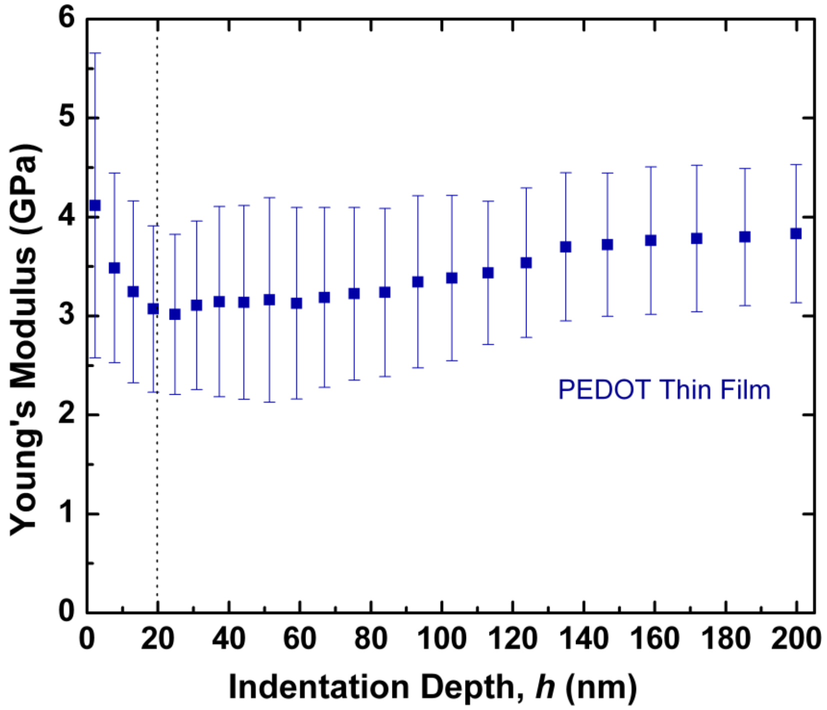

Suppl. Fig. S6.3: Nanoindentation results for an as-synthesised PEDOT film coating on glass: Young's Modulus: 3.1± 0.9 GPa, averaging over 10 indents from indentation depth of 20 to 50 nm. After 50 nm, the Young's moduli increase indicating the more significant effect from the stiffer substrate. This result is likely to be higher than the real value of the thin film, since the stiff substrate (glass) will affect the mechanical properties of the film.

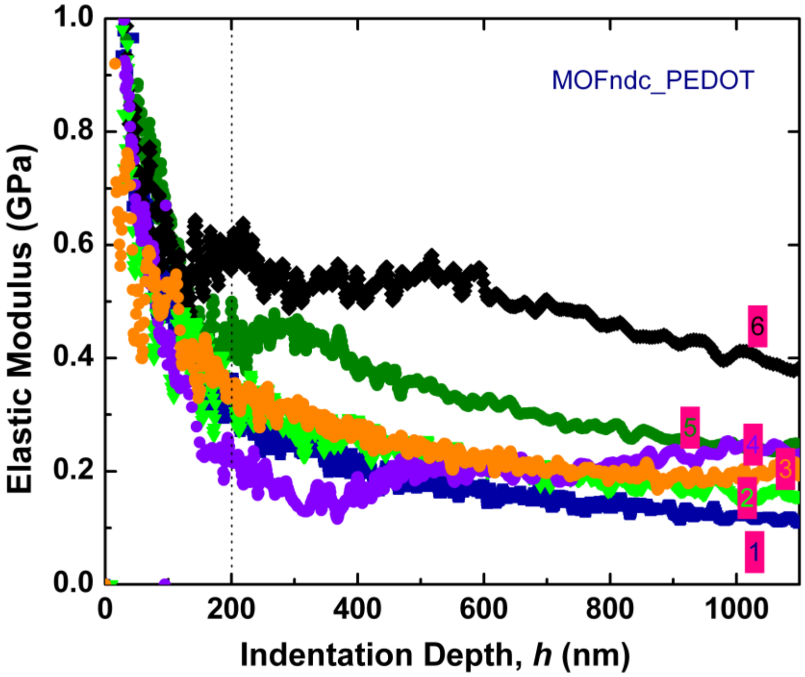

Suppl. Fig. S6.4: Nanoindentation results for PEDOT-MOF composite. A number of indentations were performed, as seen in the figure, however, the measured Young's Moduli vary between 0.1 GPa and 0.6 GPa (six individual measurements are shown in the figure), which match the Young's modulus of the PEDOT itself. Hence, we believe the high surface roughness of the composite makes the indentation results less reliable and the possible PEDOT polymer on the top surface of the PEDOT-MOF composite dominates the observed Young's Modulus.

References:

1 S. Sauerbrunn and P. Gill, *Decomposition Kinetics Using TGA*, New Castle.

2 T. Uemura, Y. Ono, K. Kitagawa and S. Kitagawa, *Macromolecules*, 2008, **41**, 87–94.

3 M. Pham, G. Vuong, F. ric-G. Fontaine and T. Do, *Cryst. Growth Des.*, 2012, **12**, 3091–3095.

4 T. Uemura, T. Kaseda and S. Kitagawa, *Chem. Mater.*, 2013, **25**, 3772–3776.

5 G. Distefano, H. Suzuki, M. Tsujimoto, S. Isoda, S. Bracco, A. Comotti, P. Sozzani, T. Uemura and S. Kitagawa, *Nat. Chem.*, 2013, **5**, 335–341.

6 S. Henke, W. Li and A. K. Cheetham, *Chem. Sci.*, 2014, 2392–2397.

7 D. N. Dybtsev, H. Chun and K. Kim, *Angew. Chemie - Int. Ed.*, 2004, **43**, 5033–5036.

8 S. Henke, A. Schneemann, A. Wütscher and R. A. Fischer, *J. Am. Chem. Soc.*, 2012, **134**, 9464–9474.

9 K. Tan, N. Nijem, P. Canepa, Q. Gong, J. Li, T. Thonhauser and Y. J. Chabal, *Chem. Mater.*, 2012, **24**, 3153–3167.

10 J. F. Arenas, J. I. Marcos, C. Granada and R. June, *Spectrochim. Acta*, 1979, **35A**, 355–363.

11 S. V. Selvaganesh, J. Mathiyarasu, K. L. N. Phani and V. Yegnaraman, *Nanoscale Res. Lett.*, 2007, **2**, 546–549.

12 A. Lisowska-Oleksiak, A. P. Nowak, M. Wilamowska, M. Sikora, W. Szczerba and C. Kapusta, *Synth. Met.*, 2010, **160**, 1234–1240.

13 D. A. Guzonas and D. E. Irish, *Can. J. Chem.*, 1988, **66**, 1249–1257.
